# The Genetic and Morphological Basis of Local Adaptation to Elevational Extremes in an Alpine Finch

**DOI:** 10.1002/ece3.72962

**Published:** 2026-01-29

**Authors:** Erica C. N. Robertson, Timothy M. Brown, Sophie Deitch, Christine M. Bossu, Erika S. Zavaleta, Mevin B. Hooten, Kristen C. Ruegg

**Affiliations:** ^1^ Department of Biology Colorado State University Fort Collins Colorado USA; ^2^ Department of Ecology and Evolutionary Biology University of California Santa Cruz California USA; ^3^ Department of Statistics and Data Sciences The University of Texas at Austin Austin Texas USA

**Keywords:** alpine, conservation genomics, F_ST_ outlier test, genome‐wide association study, local adaptation

## Abstract

Understanding patterns and mechanisms underlying local adaptation is becoming increasingly important for species conservation amid anthropogenically driven environmental change. Alpine systems are experiencing particularly intense pressure from environmental change resulting from increased rates of warming and corresponding loss of snow and ice. We integrate morphological and genetic analyses to identify traits important for local adaptation in one of the highest elevation breeding birds in North America, the Sierra Nevada Gray‐crowned Rosy‐Finch. We performed an in‐depth analysis of how traits with known links to thermoregulation in birds such as wing length, bill size, and feather microstructure vary between two populations at sites with contrasting climate and environmental conditions. We identified loci underlying these traits using a genome‐wide association study and further examined regions of the genome related to altitude adaptation and cold tolerance using F_ST_ outlier tests. Together, these results indicate that temperature, food availability, and alpine landscape features may impose multifaceted and potentially conflicting selective pressures on morphological traits important to adaptation in alpine birds. Overall, this work represents one of the first in‐depth analyses of the genetic basis of adaptation in an alpine specialist songbird.

## Graphical abstract

We investigated morphological and genetic variation in the Sierra Nevada Gray‐crowned Rosy Finch to identify traits important for local adaptation in alpine environments. By combining trait measurements with genome‐wide analyses, we uncovered loci linked to thermoregulation, altitude adaptation, and cold tolerance. Our results highlight how multiple selective pressures shape adaptation in one of North America's highest‐elevation breeding birds.

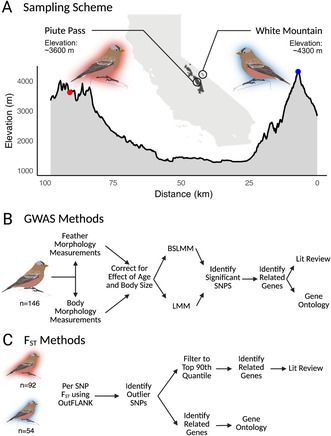

## 1 Introduction

Local adaptation occurs when natural selection favors traits that confer fitness advantages in specific habitats (Endler 1977; Kawecki and Ebert 2004; Savolainen et al. 2013). A species' adaptive capacity determines the extent to which it will be able to respond to rapid environmental change (Forester et al. 2022, 2023; Lande and Shannon 1996; Meek et al. 2023). As a result, identifying genetic and morphological traits involved in local adaptation is a key component of effective species conservation planning (Hoban et al. 2016; Meek et al. 2023). Historically, assessments of the capacity for local adaptation relied on reciprocal transplant experiments that allowed researchers to link variation in putatively ecologically important traits to differences in fitness across environmental gradients (Blanquart et al. 2013; Clausen et al. 1941; Kawecki and Ebert 2004; Savolainen et al. 2013). However, recent advances in genomic methods have made it possible to detect signatures of selection across the genome and to associate these with environmental variation and ecologically important morphological variation (Capblancq et al. 2020; Hoban et al. 2016; Lotterhos and Whitlock 2015). We leveraged recent advances in genomic methods to identify morphological and genetic traits important to local adaptation in an understudied alpine bird system.

Many montane species undergo upward range shifts with climate change (Mamantov et al. 2021). However, for those species already living near the top of a mountain system, there are constraints that may limit the ability to track their niche, a phenomenon termed the “escalator to extinction” (Freeman et al. 2018; Urban 2018). Alternatively, local adaptation can impact adaptive responses to the environment and allow species to persist despite a restricted capacity for range shifts (Aitken et al. 2008; Capblancq et al. 2020). A first step toward improving our understanding of how high‐altitude species will respond to climate change is to identify morphological traits and corresponding regions of the genome involved in adaptation to extreme alpine environments (Blanquart et al. 2013; Pritchard and Di Rienzo 2010).

Birds living in alpine regions are exposed to extreme environments throughout the year, including variable snow cover, hypoxic conditions, and dramatic temperature changes (Grabherr et al. 2010; Körner 2003). As a result, traits that contribute directly to thermoregulation are expected to be under strong selective pressure in alpine species. Previous work has shown climate‐linked traits often follow ecogeographical rules such as Bergmann's and Allen's rule. Allen's rule posits that animals adapted to colder environments will have shorter appendages than animals adapted to warm climates (Allen 1877). In birds, this rule predicts that beaks and wings will be longer in warmer environments to facilitate greater heat dissipation across a larger surface area, and shorter in colder environments to facilitate heat conservation (Greenberg et al. 2012; Lewden et al. 2023; Symonds and Tattersall 2010; Tattersall et al. 2017; Ward et al. 1999; Weeks et al. 2020, 2025). Bergmann's rule, which states that body size tends to decrease as temperature increases, has also been supported in birds (Ashton 2002; He et al. 2023). Following these rules, we predicted that birds will have shorter beaks and smaller wings as well as larger overall body sizes in higher, colder environments.

An additional aspect of beak morphology that should be investigated in the context of thermoregulation is nare length. Nares are the nostrils of the bird and are located at the top of the beak. Grinnell (1913) documented subspecific divergence in *Leucosticte tephrocotis dawsoni* , including differences in beak length and bill depth from the nostril. While nare size per se was not the focus, these measurements provide a morphological basis for interpreting variation in nasal exposure and potential heat exchange, particularly in the context of ecological differences between alpine sites. While there has not, to our knowledge, been work directly linking nare size to thermoregulatory function, this may be an important and understudied aspect of beak morphology as it relates to thermoregulation in birds. Following the logic for broader beak morphology, we can expect to see larger nares in the warmer environments to facilitate the dumping of excess heat.

Although less studied in the context of local adaptation, feather microstructure may also be important to avian species' adaptation to environmental variation in temperature. While substantial evidence suggests feather microstructure plays a key role in determining the thermoregulatory capacity of a bird's plumage (Barve et al. 2021; Stettenheim 2000; Stoutjesdijk 2003; Wolf and Walsberg 2000), there is some confusion over how environmental variation may drive feather microstructure differences (D'alba et al. 2017; Koskenpato et al. 2016; Lei et al. 2002; Pap et al. 2017, 2020). In one of the few studies that have been conducted at the population level, Koskenpato et al. (2016) found that tawny owls living in colder environments have significantly denser plumulaceous contour feathers than those living in warmer environments. This suggests that feather density may facilitate heat retention. In contrast, however, Pap et al. (2017) found that European bird species wintering in colder areas had less dense feathers than those wintering in warmer places. Thus, there is a clear need for studies specifically linking environmental variation with population‐level differences in feather microstructure.

Recent advances in population genomics have enabled the detection of loci involved in local adaptation, even in nonmodel and wild species (Faria et al. 2014). Among these, F_ST_ outlier tests are commonly used to identify genomic regions that exhibit elevated population differentiation, which may signal divergent selection (Hoban et al. 2016). However, many adaptive traits are likely polygenic, involving small effects at numerous loci (Pritchard and Di Rienzo 2010; Yeaman 2015), and such loci will not be picked up by F_ST_ outlier tests. To address this, genome‐wide association studies (GWAS) can be used to link more subtle genetic variation to phenotypic variation in ecologically important traits suspected to be under selection (Stinchcombe and Hoekstra 2008). Taken together, combining approaches that detect both population‐level divergence and polygenic trait architecture offers a powerful framework for investigating local adaptation.

**FIGURE 1:**
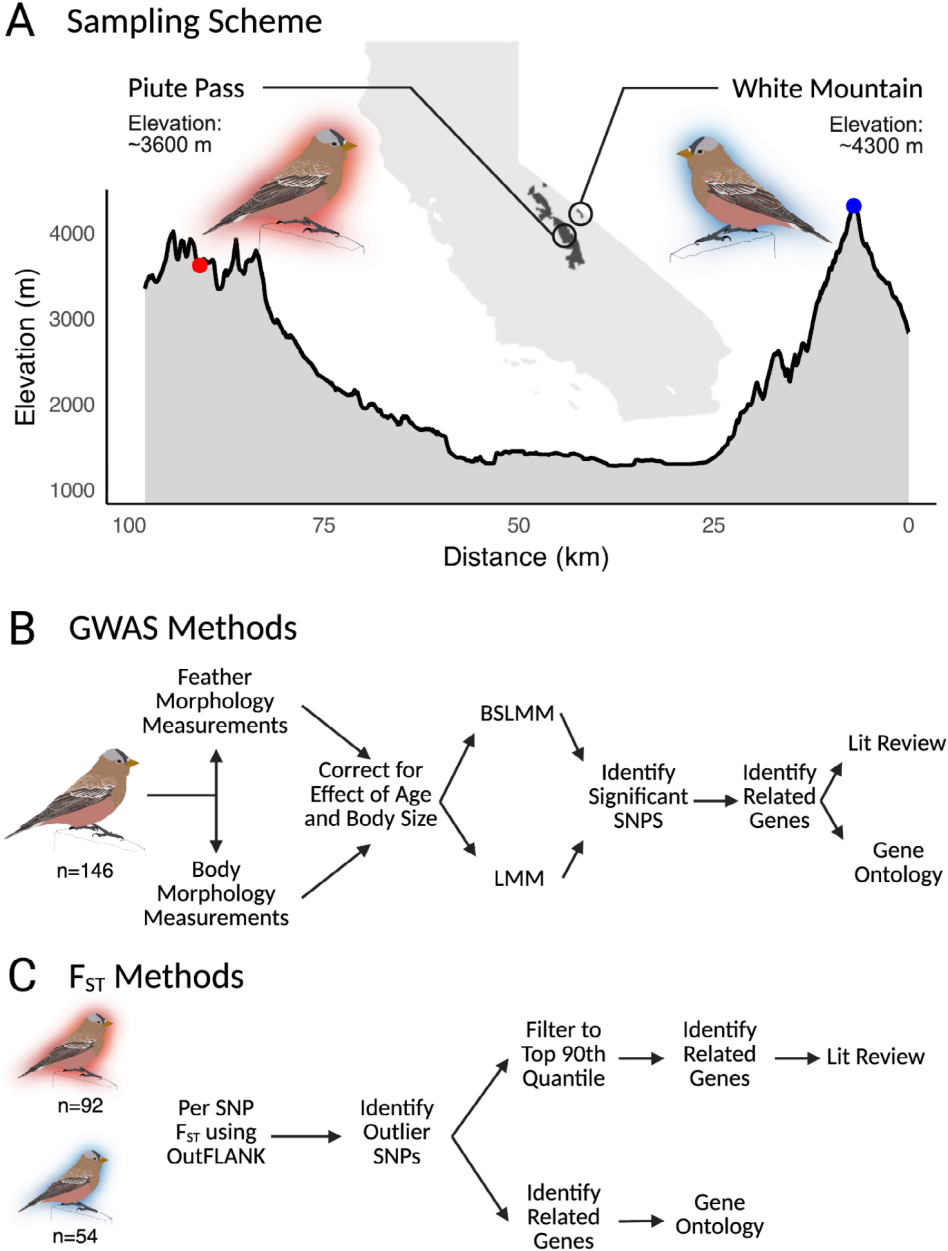
(A) Map of California highlighting the sampling location and design for two populations of Gray‐crowned Rosy‐Finch, Piute Pass and White Mountain. Elevation is noted for each site along with an elevation transect. (B) Overview of methods for GWAS analysis. Morphological measurements were corrected for the effects of sex, age, and body size before being input into BSLMM and LMM models. (C) Overview of the methods for the F_ST_ analysis. The output of both analyses were analyzed to identify significant SNPs and related genes.

We investigated the potential for local adaptation in the Sierra Nevada Gray‐crowned Rosy‐Finch ( *Leucosticte tephrocotis dawsoni* ), one of the highest‐breeding songbirds in North America. Of the roughly four groups of rosy‐finch found in North America, this subspecies is more localized, found only in California's alpine, and is a short‐distance migrant. In comparison, the broader Gray‐crowned Rosy‐Finch species range extends along the entire western edge of North America from California to Alaska. Despite long‐standing interest (Grinnell 1913, 1917; Twining 1940), research on this subspecies has been limited due to the difficulty of accessing its extreme nesting sites located on the rocky cliff faces of the Sierra Nevada and White Mountains of California, more than 3000 m above sea level. Additionally, this subspecies may act as a strong model of local adaptation due to the variation in habitat experienced within a restricted range. To help fill this knowledge gap, we conducted morphological and genomic analysis of two populations breeding at distinct places in the elevational and thermal range. Piute Pass, located in the Sierra Nevada, is lower in elevation and warmer than the White Mountains (Figure 1A). To identify traits potentially involved in local adaptation, we quantified variation in beak and feather morphology between the two populations. Based on Allen's rule, we predicted that birds in the warmer Piute Pass population would have longer wings and larger beaks. Following Bergmann's rule, we predicted that birds in Piute Pass would also have smaller overall body size. Further, we predicted that birds in the colder White Mountains location would exhibit feather traits associated with increased insulation, specifically, longer barbules and higher node density. Lastly, we used genome‐wide association studies (GWAS) to identify genes linked to feather and bill morphology and F_ST_ outlier tests to identify other potentially important sources of genetic variation (Figure 1B,C). Overall, our findings provide new insights into the process of local adaptation in an extreme alpine specialist bird and provide key baseline information on the capacity for adaptation in the face of global environmental change.

## 2 Methods

### 2.1 Site Selection

Our study compares Rosy‐Finch populations from two sites: one in the Sierra Nevada and one in the White Mountains. Sierra Gray‐crowned Rosy‐Finches occupy alpine habitats from approximately 2750–4000 m across California (Brown et al. in prep), and our study sites span a substantial portion of this range. The Piute Pass location is in the Sierra Nevada at 3470–3625 m in elevation and has a warmer average temperature during the breeding season of 8°C (with a range of 3.8°C–13.0°C). The White Mountain location, near White Mountain Peak, is higher in elevation at 4285–4344 m and colder with a mean average temperature during the breeding season of 6°C (with a range of 1.5°C–10.7°C) (extracted from AdaptWest Project 2022, see methods in Data S1 and Figure S1). Because it lies in the Sierra Nevada rain shadow, the White Mountain site receives roughly one‐third the precipitation of areas at comparable elevations in the Sierras (Rundel et al. 2008). Between the two sites, Piute Pass receives more precipitation as snow and has a more persistent snowpack than the White Mountain location. Additionally, aquatic environments such as streams, lakes and ephemeral pools are more abundant in the Sierra Nevada than the White Mountains (Rundel et al. 2008). The alpine ecosystems of these mountains can be delineated as communities occurring above tree line (Rundel and Millar 2016). Environmental stressors such as extreme winter temperatures, short growing seasons, high winds, low partial pressures of O_2_, and limited water availability are characteristic of these alpine environments (Grabherr et al. 2010; Rundel and Millar 2016). Separating the western Sierra Nevada Mountains and the eastern White Mountains is the Owens Valley, a subregion of the Great Basin Desert (Belnap et al. 2016).

### 2.2 Sample Collection

A total of 171 Sierra Nevada Gray‐crowned Rosy‐Finches were captured using potter traps at White Mountain (*n* = 98) and Piute Pass (*n* = 73) in June and July, the peak of the species' breeding season (MacDougall‐Shackleton et al. 2000). Captures within each mountain range occurred across multiple locations within approximately 1 km^2^ at each site (White Mountain or Piute Pass) to maximize sample sizes while maintaining site‐specific environmental characteristics. The sampling thus, in total, represents only these two sites. Not all morphology was able to be collected for every bird, resulting in slight variation in the types of data collected for each individual (see details in supplemental methods, see sample sizes for each trait in Table S1). Blood samples were collected from 150 of the captured birds using the brachial wing vein and stored in Queen's lysis buffer at room temperature (Owen 2011; Seutin et al. 1991). At the same time, morphological measurements—tarsus length, wing chord, beak width, beak length, beak depth, nare length, and mass—were also collected from 154 of the birds using an electronic caliper and 5–10 body feathers were collected from the breast of all individuals. Additionally, birds were aged and sexed following the guidelines established in Pyle (2022) (see supplemental methodology). Birds were banded to allow for identification of the individuals and recognize recaptures, then released. All handling and banding of birds was done following the guidelines and protocols of the US Geological Survey (USGS) Bird Banding Laboratory (BBL) and complied with permits and permissions from federal and state agencies. All measurements were taken by two researchers (T.B.M. and E.S.Z.), with one (T.B.M.) collecting approximately 80% of the data. Because the majority of measurements were taken by a single observer, and all followed the same standardized protocol, observer identity was not included as a covariate in subsequent analyses.

### 2.3 Feather Microstructure Measurements

We measured the microstructure of three feathers for each individual. For each feather, three photographs were taken using an Olympus BX51 Microscope: One photo each of an unbroken pennaceous and plumulaceous barb from the center of each region was taken at 4× objective, and one photo of a plumulaceous barbule was taken at 10× objective (Figure 3A). Structures were then measured from the photos using the segmented line tool in ImageJ. For the pennaceous and plumulaceous barbule length, we measured one barbule from each region starting from the distal tip to where the barbule meets the barb. For the pennaceous and plumulaceous barbule density, we drew a 0.5 mm line along the middle of a barb, counting the number of barbules along that line. And finally, for plumulaceous node density, we drew a 0.2 mm line along the center of a barbule, counting the number of nodes along that line. Each replicate measure for a feather trait was averaged per individual prior to downstream analysis. Approximately 95% of feathers were measured by one researcher (SD), and the observer was not included as a covariate in subsequent analyses.

### 2.4 Statistical Analysis for Local Adaptation

**FIGURE 2:**
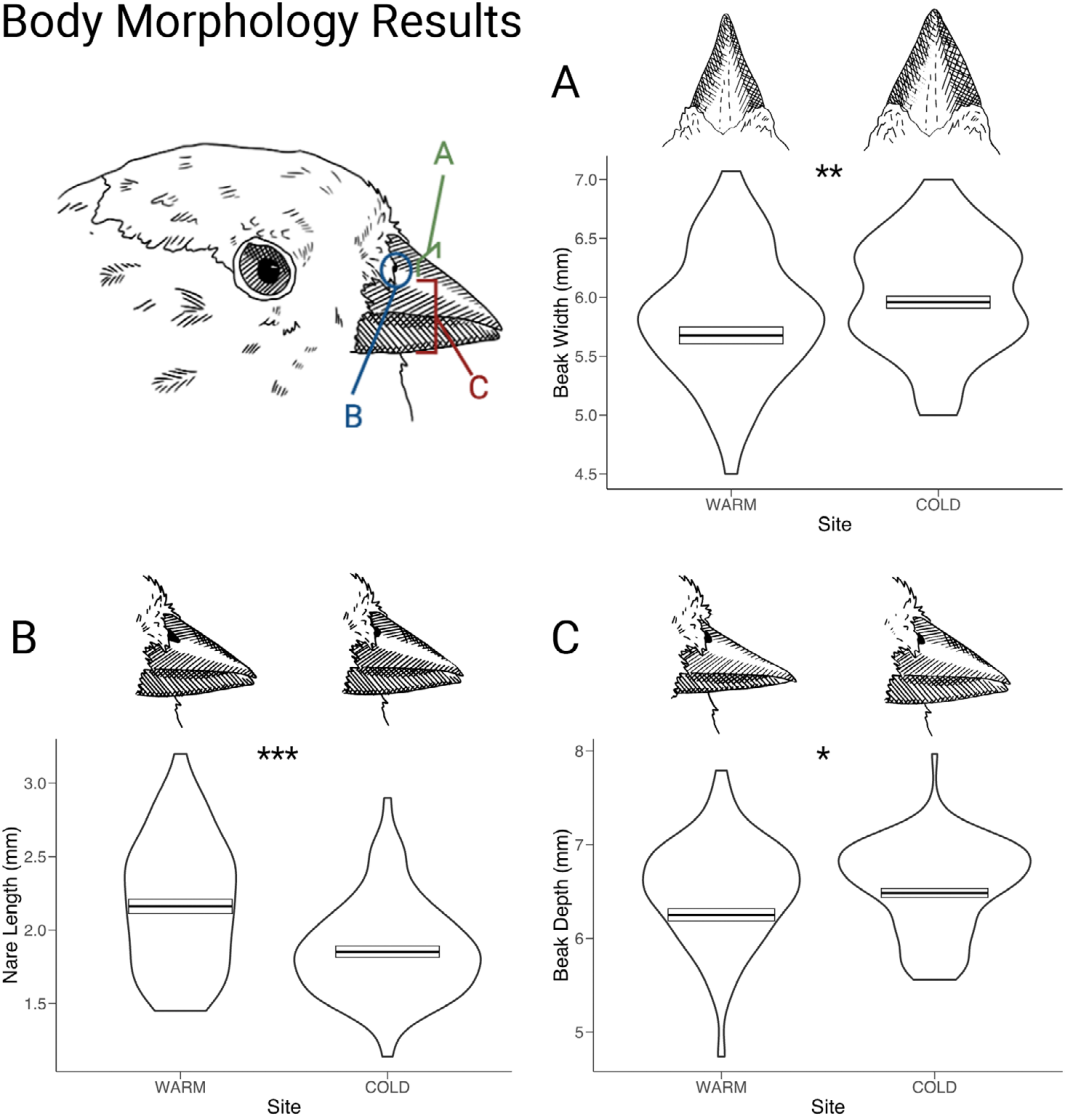
Violin plots of beak morphology raw measurement: (A) beak width, (B) nare length, and (C) beak depth. Overlaid are the corrected mean trait measurements shown with a crossbar. Significance of the effect of site on the morphological trait is shown for each comparison. Piute Pass is the warm site and White Mountain is the cold site. We found that nares were longer while beak widths and depths were smaller in the warmer environment.

**FIGURE 3:**
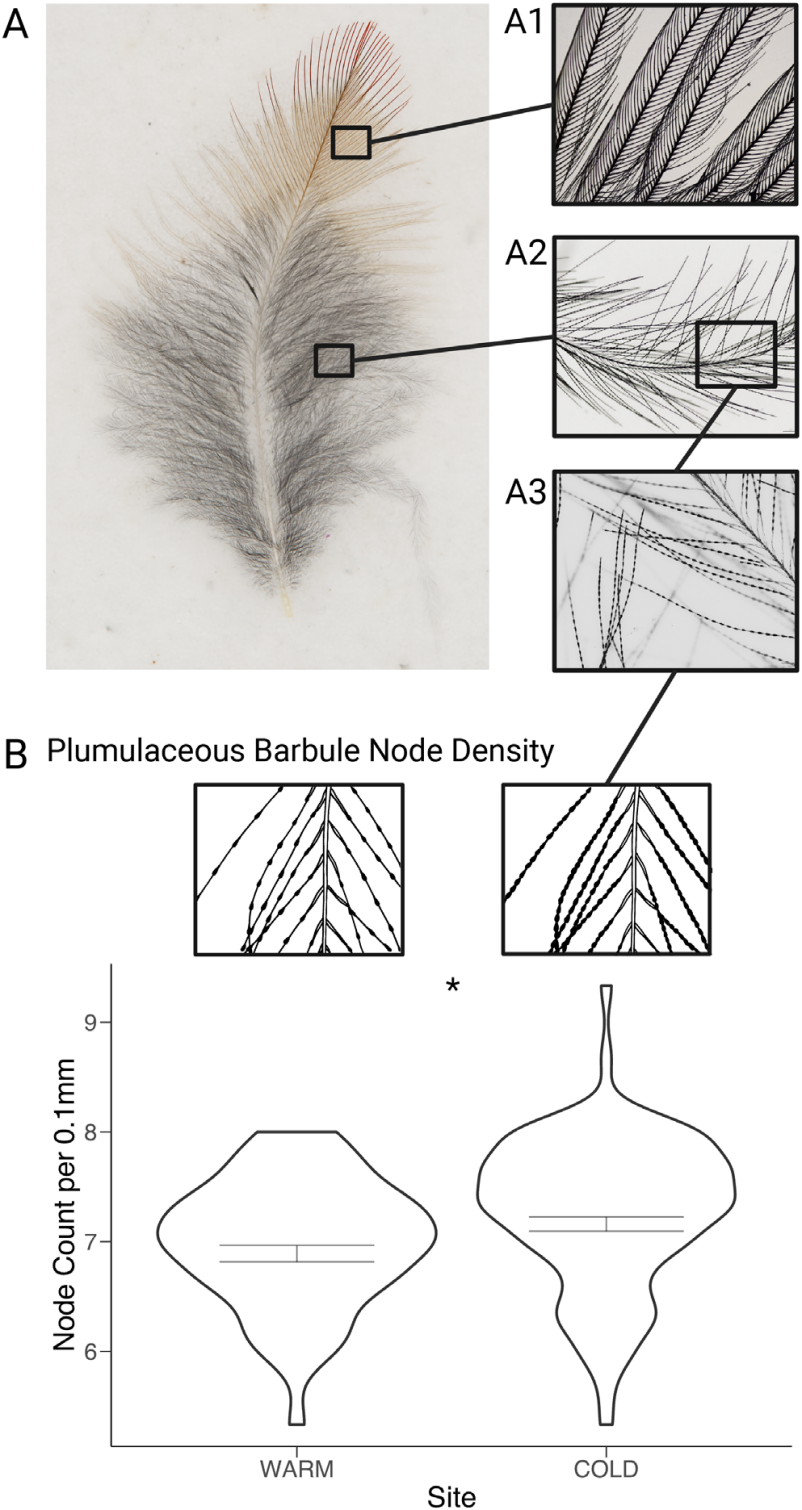
(A) Photograph showing a body feather from a Sierra Nevada Gray‐crowned Rosy‐Finch captured at White Mountain. Highlighted is a microscopic image of the pennaceous and plumulaceous barbule structure. (B) Violin plots of node density raw measurements, with illustrations above of the structure of plumulaceous barbule nodes. Overlaid on the plots are the corrected mean trait measurements shown with a crossbar. Significance of the effect of site on the morphological trait is shown for each comparison.

Body size and sex can confound morphological comparisons among individual birds (D. C. Adams et al. 2020). To account for these effects in our investigation of Allen's rule, we included sex and tarsus length as predictor variables in our models. We used tarsus length as an index of body size because it correlates strongly with mass but is less affected by short‐term fluctuations in condition; this approach has been used in recent large‐scale morphological studies (Demery et al. 2021; Weeks et al. 2020; Zimova et al. 2023). As additional validation of tarsus as a proxy size for body size, we generated a PC axis that incorporated multiple allometric traits and found that this was highly correlated (0.7) with tarsus (see supplement for additional details). We proceeded with tarsus as our proxy for body size in all further analyses. To assess trait variation between sites, we used both univariate and multivariate approaches (see Tables S2 and S3 for a summary of trait values). We began by constructing linear models with tarsus length, sex, age, and site code as predictors and used AIC weights to identify the combination of variables that best explained trait variation. Traits for which site code was a significant predictor were considered significantly different between the two populations. We opted not to use a Bonferroni correction for the univariate models as each model had a specific hypothesis associated with it. We used *emmeans* (Lenth 2025) to calculate average trait values while controlling for predictors other than site code, and these adjusted means were overlaid on the raw data in a violin plot (Figures 2 and 3B). To test our hypotheses derived from Bergmann's rule, we compared both tarsus length and a principal component axis representing overall body size using linear models. Each model included age and sex as covariates to account for potential confounding effects on trait variation. We then assessed whether body size differed significantly between the two populations by including site as a predictor.

We also conducted a multivariate analysis to account for potential correlations among traits in our investigation of Allen's rule. For our multivariate approach, we incorporated all body morphology traits (wing chord, nare length, beak depth, beak width, beak length) into a MANOVA model with site, age, sex, and tarsus as predictors. The same analysis was performed with all of the feather traits (pennaceous barbule density and length, plumulaceous barbule density, length, and node density) together.

### 2.5 Sequencing and Genotyping

DNA was extracted from blood samples of 150 individuals using Qiagen's DNeasy Blood and Tissue Extraction Kit protocol and quantified with the Qubit dsDNA HS Assay Kit. We used a modified version of Illumina's Nextera Library Preparation protocol to prepare WGS libraries and pooled the libraries by equal mass before sequencing. The resulting libraries were sequenced on 2 lanes of an Illumina NovaSeq 6000 at Novogene Corporation.

To process the raw sequence reads and detect variants, we utilized Snakemake (Mölder et al. 2025), a workflow management system that provides efficiency, adaptability, and reproducibility. The pipeline we adapted to our species can be found on Github (https://github.com/eriqande/mega‐non‐model‐wgs‐snakeflow/). To summarize the workflow, sequence data were trimmed using fastp (S. Chen et al. 2018) to remove adaptor sequences and polyG tails using a sliding window, and then aligned to a Brown‐capped Rosy‐Finch ( *Leucosticte australis* ) reference genome (GenBank: GCA_025504685.1) using Burrows‐Wheeler Aligner software (H. Li and Durbin 2009). We marked PCR duplicates using SAMtools (Danecek et al. 2021) and read groups (sample, lane, library) were added using Picard (http://broadinstitute.githut.io/picard). Individual coverage was estimated using SAMtools, and, given the range of coverage (7.4–19X), we downsampled bam files to 10× using the SAMtools subsample function (Danecek et al. 2021). Individual gvcf files were created using the GATK HaplotypeCaller (Poplin et al. 2018) with high base quality score filters (‐‐min‐base‐quality‐score 33 ‐‐minimum‐mapping‐quality 20) to remove batch effects (Lou and Therkildsen 2022). We then parallelized the calling of genotypes across 3 million bp regions across the genome using the GenomicsDBImport and GenotypeGVCFs functions (der Auwera and O'Connor 2020; McKenna et al. 2010). GATK versions above 4.0 call missing data as homozygous reference, which can bias downstream analysis. To avoid this, all loci with a depth of zero were manually marked as missing. To remove systematic errors according to GATK variant quality score recalibration (VQSR) best practices, we hard filtered variants with the following parameters: StrandOddsRatio (> 3.0), FisherStrand (> 60.0), MappingQuality (> 40.0), and Quality by Depth (> 2.0). We further filtered for single nucleotide polymorphisms (SNPs), removing indels, filtering for missingness (< 80%), allele frequencies (> 0.05 and < 0.95), depth (> 4×), quality (> 30.0) using BCFtools (Danecek et al. 2021). After filtering, the resulting high‐quality SNPs were passed through BEAGLE 4.1 (Browning and Browning 2016) to impute missing genotypes.

### 2.6 Population Genetic Structure Analysis

Population structure was assessed using a Principal Component Analysis (PCA) followed by ADMIXTURE (Alexander et al. 2009). SNPs identified from sequence data were pruned for linkage disequilibrium in PLINK (Purcell et al. 2007) using a 50 kb window, 10 SNP window step size, and an *R* ^2^ threshold of 0.2. Eigenvalues for the PCA were then generated based on the pruned SNPs using PLINK, and the first two principal components (PC) were plotted in R. To further confirm a lack of population structure, ADMIXTURE was used to test *k* = 1–3. The results were plotted using R, and the cv values were evaluated.

### 2.7 Genome‐Wide Association Study

Two GWAS models were implemented on all traits using GEMMA (Zhou et al. 2013): a Bayesian sparse linear model, BSLMM, and a univariate linear model, ULMM. These GWAS approaches allow us to detect SNPs associated with a given trait while explicitly accounting for population structure and relatedness through the incorporation of random effects whose correlation structure is characterized by a relatedness matrix. Input files were generated with PLINK (Purcell et al. 2007). We implemented a Bayesian sparse linear mixed model (BSLMM) using 500,000 MCMC burn‐in iterations that were discarded and 5 million MCMC iterations that were saved. We then filtered for SNPs that had a mean posterior inclusion probability (PIP) above 0.01. A univariate linear mixed model (ULMM) employing the Wald test was also conducted (see Table S7 for λGC values for each model and Figure S6 for PP plots for each trait). The Wald test assesses the significance of the SNP genotypes in a linear regression model and is used to test the null hypothesis that the effect size of a genetic variant on the phenotypic trait is zero (i.e., there is no association between the variant and the trait). SNPs were then filtered based on a *p* value threshold of 5 × 10^−8^, a threshold widely used in GWAS studies (e.g., Chen et al. 2021).

### 2.8 Genome Wide F_ST_

To estimate per site F_ST_, we used OutFLANK (Whitlock and Lotterhos 2015). OutFLANK identifies outlier F_ST_ SNPs by modeling the distribution of F_ST_ values across loci using a trimmed likelihood approach to exclude loci under strong selection. It then fits a chi‐squared distribution to the neutral F_ST_ values and identifies SNPs with unusually high or low F_ST_ as potential outliers, suggesting loci under selection. We used a false discovery rate threshold of 0.005 to determine outlier SNPs. We took the 90th quantile of these F_ST_ values to narrow down the top SNPs for literature review and plotted these on a Manhattan plot. This allowed for isolation of the most divergent loci as well as easier visualization. We then determined the F_ST_ values for the SNPs previously identified in the GWAS analyses based on the values calculated by OutFLANK.

### 2.9 Identification of Associated Genes, Enrichment Analysis, and Literature Review

To identify genes associated with significant SNPs from our GWAS and F_ST_ analysis, we implemented BEDTools (Quinlan and Hall 2010) closest function with a previously created Brown‐capped Rosy‐Finch gene annotation (Funk et al. 2023) to identify genes close to the SNP positions. The output from this tool was filtered to find only protein coding genes within 25 kb of the given SNP. For plotting purposes, we used the NUCmer function in MUMmer4.x (Marçais et al. 2018) to align the Brown‐capped Rosy‐Finch genome assembly to the Zebra Finch ( *Taeniopygia guttata* ) genome assembly (GenBank: GCF_003957565.2). We then filtered the delta alignment output of NUCmer to keep only matches that were greater than 400 bps and used show‐coords to display the coordinates. We then used custom R scripts to convert our scaffold and positions to Zebra Finch chromosome positions for plotting SNPs in Manhattan plots.

Enrichment in gene ontology (GO) terms was performed with Panther 19.0 (Thomas et al. 2022). For this analysis, *Gallus gallus* was used as the background gene set. Enrichment thresholds were set to *p* < 0.05 after Bonferroni correction. Gene network analysis was conducted using STRING (Szklarczyk et al. 2023) with zebra finch as the organismal reference for background genes. STRING (Search Tool for the Retrieval of Interacting Genes/Proteins) is a database that predicts and visualizes protein–protein interactions based on known and predicted associations from experiments, literature, and computational methods (Szklarczyk et al. 2023). For F_ST_ genes, all genes associated with the OutFLANK outliers were input into enrichment analyses. Genes resulting from the GWAS analysis were grouped with all beak morphology trait genes run as a single analysis, all feather trait genes run together, and wing chord genes run in another analysis.

We performed a literature search for all of the candidate genes using google scholar and the search terms gene name followed by, bird, trait specific term (when applicable), environmental adaptation, local adaptation (e.g., “‘XIAP’ gene beak” or “‘DSCAM’ gene environmental adaptation”). For F_ST_ SNPs, only those genes associated with the 90th quantile of SNPs were evaluated in the literature. Genes were grouped by their key functions or associations as described in the literature.

## 3 Results

### 3.1 Local Adaptation of Body and Feather Morphology

For the univariate approach, AIC model selection indicated five traits for which site was a significant predictor. For wing chord, the best model included tarsus length, age, sex, and site. The model showed high explanatory power (adjusted *R* ^2^ = 0.673, *p* < 0.001) and showed that birds at the White Mountain location had shorter wing chords compared to Piute Pass (PIPA) (*p* = 0.003). The best model for nare length included tarsus, sex, and site and had lower explanatory power (adjusted *R* ^2^ = 0.197) but was still highly significant (*p* < 0.001). This model found that nare length was shorter in the White Mountain population (*p* < 0.001). Similarly, beak width had tarsus, age and site as significant predictors (adjusted *R* ^2^ = 0.135, *p* < 0.001) with White Mountain having beaks that are wider than those in Piute Pass (*p* < 0.001). Beak depth had tarsus, age and site as the best predictors (adjusted *R* ^2^ = 0.250) and found that White Mountain has significantly deeper beaks than Piute Pass (*p* = 0.003). Beak length was not significantly different between the two locations and was best predicted by sex (adjusted *R* ^2^ = 0.516, *p* < 0.001). For feather microstructure, the only trait that had site as a significant predictor was plumulaceous node density. This model included tarsus and age as well as site but had low explanatory power (adjusted *R* ^2^ = 0.076, *p* < 0.001). It showed that plumulaceous node density was higher in the White Mountain populations (*p* < 0.001). The best model for plumulaceous barbule length based on AIC weights included site as a predictor; however, the effect size was uncertain, and the effect of site was not statistically significant (adjusted *R* ^2^ = 0.142, *p* = 0.078). Pennaceous barbule density had tarsus and age as the best predictors (adjusted *R* ^2^ = 0.365, *p* < 0.001), pennaceous barbule length had sex as the best predictor (adjusted *R* ^2^ = 0.307, *p* < 0.001), and plumulaceous barbule density had tarsus and age as the best predictors (adjusted *R* ^2^ = 0.076, *p* = 0.004). We found that body size did not vary between sites. In our model using tarsus (adjusted *R* ^2^ = 0.165, *p* < 0.001) both age and sex were significant predictors (*p* < 0.001 and *p* < 0.05); however, site was not significant (*p* = 0.417). Our comparison using the PC axis representing size (adjusted *R* ^2^ = 0.4639, *p* < 0.001) had similar results with age and sex as significant and site as not (*p* = 0.53).

When looking at the results of the multivariate analysis, we see that for body morphology traits site was a highly significant explanatory variable (*p* = 1.327e‐11). Tarsus, age, and sex were also highly significant (*p* < 2.2e‐16). The MANOVA on feather traits also had site as a significant predictor, although the *p* value was less significant (*p* = 0.036). Age was also significant (*p* = 1.5e‐14), but tarsus and sex were not (*p* > 0.05). For details on the models tested, models chosen, and full model results, see Tables S4–S6.

### 3.2 Evaluation of Population Structure

Whole‐genome sequencing was performed on 150 individuals and 146 were included in downstream analysis. Four individuals were removed because their collection location could not be validated. Variant filtering resulted in 7,739,836 SNPs for subsequent genetic analysis. Linkage pruning removed 5,526,104 SNPs. Visualizing PLINK PCA results revealed little population structure with most individuals clustering together (Figure S2). ADMIXTURE results confirmed that there is no population structure in these samples because *k* = 1 had the lowest cv error (0.599) compared to *k* = 2 or 3 (0.612 and 0.627).

### 3.3 Genes Identified With Genome‐Wide Association Approach

**FIGURE 4:**
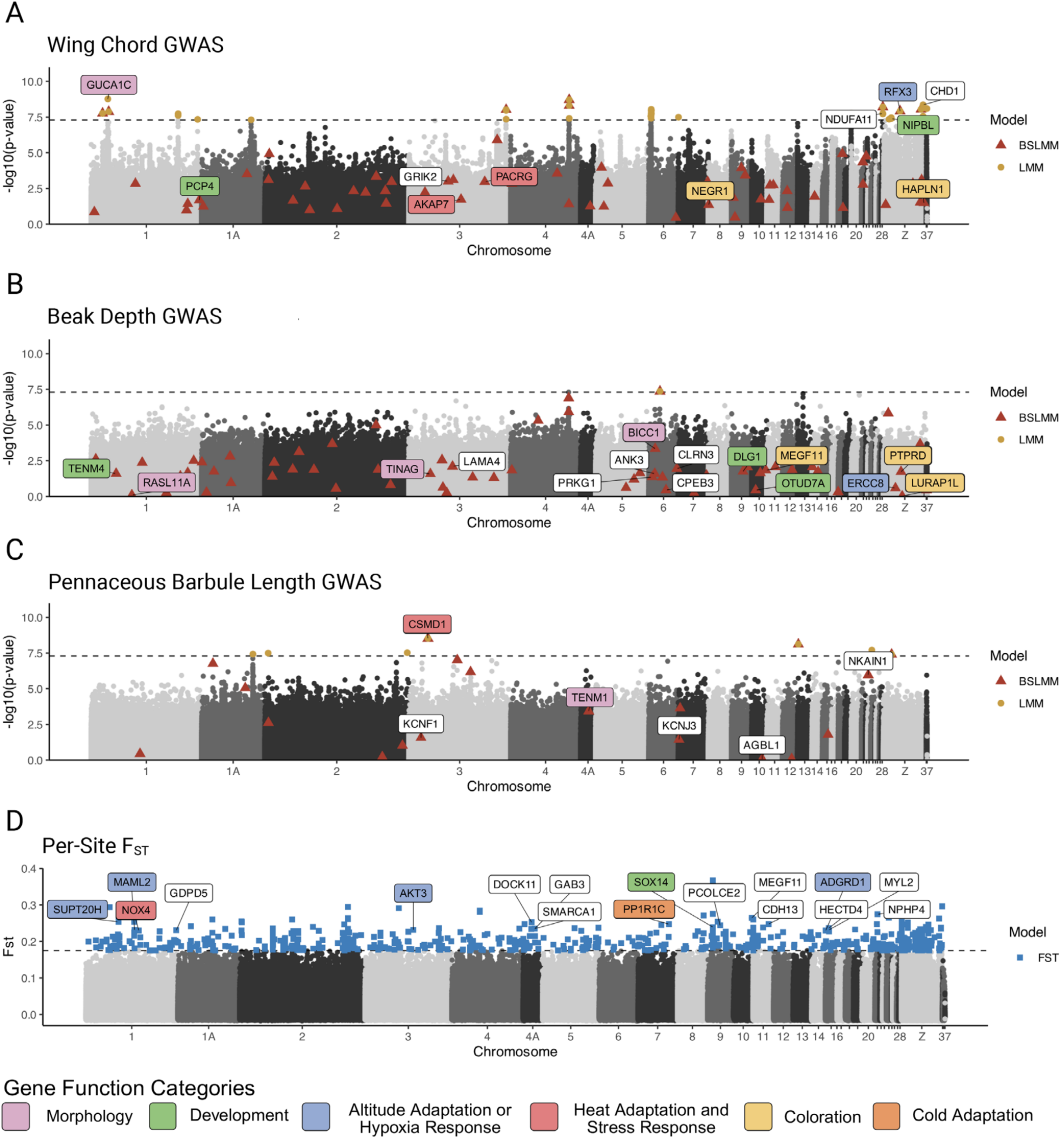
Manhattan plot depicting the results of the LMM analysis on beak depth (A), wing chord (B), and pennaceous barbule length (C) with –log_10_(*p*‐value) on the *y*‐axis and the mapped Zebra Finch chromosome location on the *x*‐axis. Significant SNPs from the BSLMM analysis are overlaid onto the LMM results, plotted with their corresponding *p* values, and corresponding genes are noted. Also plotted are the results of the per‐site F_ST_ results (D), using the 90th quantile threshold of 0.235. Corresponding genes are also shown, and functional categories are highlighted by color category. See Table 1 for more functional details.

The univariate linear mixed model identified a total of 64 SNPs for wing chord, beak length, beak width, pennaceous barbule length, and pennaceous barbule density. Using a Brown‐capped Rosy‐Finch gene annotation, we identified a total of 26 unique genes corresponding to these SNPs (Table 1). We filtered out genes that were not fully characterized (e.g., “LOC100221041”), leaving 14 genes. The Bayesian sparse linear mixed model identified 266 SNPs for wing chord, nare length, beak length, depth and width, pennaceous barbule length and density, and plumulaceous barbule length and node density. We identified 146 genes associated with these SNPs (Table 1), 110 of which had been fully characterized. For each trait, the global view of *p* values for all SNPs and associated genes across both models are represented by Manhattan plots (Figure 4A–C, Figures S3–S5). Given the sample size (*n* = 146), this GWAS is best viewed as conservative, detecting only loci with relatively strong effects on morphology.

**TABLE 1:** Summary of GWAS results by trait. Shown are the genes for which functional information was available.

Trait	Gene	Model	Function	Details	References
Wing chord	PCP4	BSLMM	Development, stress response	Bone development, stress response in quail	Xiao et al. (2008), Steven (2021)
Wing chord	GRIK2	BSLMM	Reproduction	Egg quality and production in goose and duck	Gao et al. (2023), Bhavana et al. (2022)
Wing chord	AKAP7	BSLMM	Stress response	Stress response in quail	Steven (2021)
Wing chord	PACRG	BSLMM	Heat stress	Heat stress response in chickens	Tian et al. (2020)
Wing chord	HAPLN1	BSLMM	Coloration	Plumage coloration in ducks	Zhang et al. (2024)
Wing chord	NEGR1	BSLMM	Development, coloration	Neural development, plumage coloration in crested ibis	Sun et al. (2020)
Wing chord	PGLYRP2	BSLMM/LMM	Immunity, heat stress	Immune response in vertebrates, heat stress response in chickens	Ahmad et al. (2020), Kim et al. (2021)
Wing chord	GUCA1C	LMM	Morphology	Visual systems in birds, bone quality in ducks, wing deformity in chickens	Wu et al. (2021), Gesemann and Neuhauss (2023), Li et al. (2020), Mohammadi (2024)
Wing chord	RFX3	LMM	Hypoxia adaptation	Altitude adaptation in wolves	Zhang et al. (2014)
Wing chord	NIPBL	LMM	Development, DNA repair	Developmental regulation and DNA repair	Barnes et al. (2019)
Wing chord	CHD1	LMM	Gene regulation	Modulates gene expression in birds	Fridolfsson and Ellegren (2000)
Wing chord	NDUFA11	LMM	Metabolism, respiration	Respiratory system support in buffalo, mitochondrial activity in songbirds	Clark et al. (2024), Sadeesh et al. (2025)
Beak depth	TENM4	BSLMM	Development, coloration, reproduction	Neural development in quail, dorsal feather coloration in duck, breeding timing in great tit	Twumasi et al. (2024), Steven (2021), Gienapp et al. (2017)
Beak depth	RASL11A	BSLMM	Morphology	Craniofacial development in buteo	Abernathy (2021)
Beak depth	TINAG	BSLMM	Morphology	Beak development in chicken	Bai et al. (2018)
Beak depth	LAMA4	BSLMM	Migration	Migration timing in Swainson‚Äôs thrush	Johnston et al. (2016)
Beak depth	LURAP1L	BSLMM	Coloration	Pigmentation in mallards	Ma et al. (2021)
Beak depth	PTPRD	BSLMM	Communication, coloration	Vocal signaling in flycatchers, pigmentation in mallards	García et al. (2023), Ma et al. (2021)
Beak depth	CLRN3	BSLMM	Reproduction, sex differentiation	Sex differentiation in chicken	Luo et al. (2024)
Beak depth	CPEB3	BSLMM	Reproduction	Oocyte meiosis regulator in chicken	Chen et al. (2022)
Beak depth	BICC1	BSLMM	Morphology, body size	Bone development in chicken and mice, body size in chicken and hermit thrush	Kinsella et al. (2019), Johnsson et al. (2015), Mesner et al. (2014)
Beak depth	ANK3	BSLMM	Migration	Migration in birds	Lennon (2022)
Beak depth	PRKG1	BSLMM	Reproduction, behavior, coloration	Ovarian function in duck, foraging behavior in humans, signal transduction and feather pigmentation in ducks	Sun et al. (2024), Struk et al. (2019), Twumasi et al. (2024)
Beak depth	OTUD7A	BSLMM	Development	Neural development and adaptation to urban environments in bananaquit	Mascarenhas et al. (2023)
Beak depth	ERCC8	BSLMM	Hypoxia adaptation	Altitude adaptation in lizard	Yan et al. (2022)
Beak depth	MEGF11	BSLMM	Coloration	Dorsal feather coloration in duck	Twumasi et al. (2024)
Beak depth	DLG1	BSLMM	Development, regulation	Embryo development in mice, regulatory development in birds	Seki et al. (2017)
Nare length	NRIP1	BSLMM	Reproduction, adaptation, metabolism	Female reproduction in goose, nocturnal adaptation in owls, mitochondrial biosynthesis in vertebrates	Zheng et al. (2023), Cho et al. (2019), da Costa Moreno (2022)
Nare length	ATP1A1	BSLMM	Migration	Migration in birds	Lennon (2022)
Nare length	TENM4	BSLMM	Development, coloration, reproduction	Neural development in quail, dorsal feather coloration in duck, breeding timing in great tit	Twumasi et al. (2024), Steven (2021), Gienapp et al. (2017)
Nare length	KALRN	BSLMM	Neurodevelopment	Cognitive ability in chickadee	Ricchetti et al. (2024)
Nare length	CNTNAP5	BSLMM	Development, memory, hypoxia adaptation	Brain development and signaling in zebra finch, spatial memory in chickadees, hypoxia response in chickens	Gilbert (2021), Semenov et al. (2024), Li and Durbin (2009)
Nare length	KCTD18	BSLMM	Immunity, communication	Immune response in vultures, vocal learning in chickens	Chung et al. (2015), Lovell et al. (2013)
Nare length	PLPP3	BSLMM	Morphology, metabolism	Culmen length in buteo	Abernathy (2021)
Nare length	DLG1	BSLMM	Development, regulation	Embryo development in mice, regulatory development in birds	Seki et al. (2017)
Nare length	ADAMTS18	BSLMM	Morphology, bone density	Bone mineral density in humans and chickens	Xiong et al. (2009), Guo et al. (2017)
Nare length	PSD2	BSLMM	Coloration, stress response	Melanin production in owls, stress response in quail	Ducrest et al. (2025), Steven (2021)
Nare length	BRAT1	BSLMM	Stress response	Stress response in chickens	Rodriguez et al. (2025)
Pennaceous barbule length	KCNF1	BSLMM	Coastal adaptation	Coastal adaptation in song sparrow and frogs	Clark et al. (2024)
Pennaceous barbule length	CSMD1	BSLMM/LMM	Heat stress, hypoxia adaptation	Heat adaptation in chickens, adaptation to altitude in humans	Bai et al. (2024), De Loma et al. (2025)
Pennaceous barbule length	NKAIN1	BSLMM	Immunity, migration	Immune response in house finch, migration tendency in European blackcap	Kuttiyarthu Veetil et al. (2024), Delmore et al. (2020)
Pennaceous barbule length	KCNJ3	BSLMM	Reproduction	Egg production in duck	Sun et al. (2024)
Pennaceous barbule length	AGBL1	BSLMM	Metabolism	Fatty acid composition in chicken	Cho et al. (2023)
Pennaceous barbule length	TENM1	BSLMM	Morphology	Craniofacial development in pigeons	Boer et al. (2021)
Plumulaceous node density	EFNA2	BSLMM	Heat stress, morphology, coloration	Heat stress response in chickens, bone morphogenesis and remolding in ground tit, color differences in bulbul	Zhuang et al. (2019), Cheng et al. (2020), Shakya (2020)
Plumulaceous node density	STK10	BSLMM	Morphology	Cranium and bill depth in buteo	Abernathy (2021)
Plumulaceous barbule density	BORCS6	BSLMM	Metabolism	Meat quality in chickens	Alsoufi et al. (2023)
Plumulaceous barbule density	HSF2	BSLMM	Heat stress	Response to heat stress in chickens	Xie et al. (2014)
Plumulaceous barbule density	PRKN	BSLMM	Domestication	Domestication in birds	Farias‐Virgens et al. (2025)
Plumulaceous barbule density	NRXN1	BSLMM	Neurodevelopment, communication, coloration	Neural connections, plumage coloration in crested ibis, vocal rhythm in birds, speech disorders in humans	Sun et al. (2020), Sebastianelli et al. (2024)
Plumulaceous barbule density	ALDH1A1	BSLMM	Hypoxia adaptation, metabolism, vision	Hypoxia adaptation and fat metabolism in rosy finch, eyesight in owl and mice	Bao et al. (2016), Funk et al. (2023), Borges et al. (2019)
Plumulaceous barbule density	ADAMTS19	BSLMM	Morphology	Rumpless trait in chickens	Chen et al. (2024)
Plumulaceous barbule density	HERC4	BSLMM	Coloration, migration	Plumage color in duck, migration in European blackbirds	Zhang et al. (2023), Franchini et al. (2017)
Plumulaceous barbule density	SMC4	BSLMM	Development	Feather follicle development in goose	Hu et al. (2020)
Plumulaceous barbule density	SPAG16	BSLMM	Reproduction, coloration, longevity	Sexual development in birds‐of‐paradise, plumage coloration in ducks, longevity in mammals	Prost et al. (2018), Zhang et al. (2024), Matsuda and Makino (2024)

GO term analysis using Panther produced significant results for beak depth and a combined set of all feather traits. For beak depth, the top terms were presynaptic membrane assembly (*p* = 1.44 × 10^−5^), synaptic membrane adhesion (specifically cell adhesion) (*p* = 1.01 × 10^−5^), and heterophilic cell–cell adhesion via plasma membrane cell adhesion molecules (*p* = 1.13 × 10^−5^). For the feather traits (plumulaceous barbule and node density combined with pennaceous barbule length), fructosamine catabolic process was a significant term (*p* = 1.12 × 10^−6^). Wing chord had no significant GO terms. STRING analysis for beak and feather traits, as well as wing chord, did not find significantly more interactions than expected.

### 3.4 Genes Identified Through High Fixation Index (F_ST_ ) Between the Two Populations

Using OutFLANK, 831 loci were identified as having significantly high F_ST_ when comparing the White Mountain and Piute Pass populations, corresponding to 265 genes (Figure 4D). No GWAS SNPs were flagged as having significant F_ST_ values. The minimum F_ST_ to be considered an outlier was 0.17513. The mean F_ST_ of SNPs not considered outliers was 0.0045. Using a 90th quantile, we determined F_ST_ = 0.235 to be the threshold, narrowing down the SNPs to 105 loci. For the smaller set of loci, bedtools identified 61 related genes (Table 2). We ran STRING and GO term enrichments on both the larger and smaller set of genes. The smaller set did not have a significant number of connections over all or any significantly enriched GO terms. The larger set of 264 genes, however, had an overall significant number of connections (*p* < 0.05) and had multiple significant annotated keywords (sourced from UniProt). The most significant was the keyword repeat (FDR < 0.001); the next was ubl conjugation pathways (FDR < 0.05).

**TABLE 2:** Summary of F_ST_ outlier results. Shown are the genes for which functional information was available, from the narrower (90th quantile) F_ST_ results.

Gene	Function	References
GDPD5	Lipid metabolism	Feng et al. (2023)
NOX4	Heat stress, altitude adaptation	Goel et al. (2021), Diebold et al. (2010)
MAML2	Altitude adaptation	Schweizer et al. (2021)
SUPT20H	Altitude adaptation	Schweizer et al. (2021)
PPP1R1C	Cold adaptation	Fedorova et al. (2022)
SOX14	Feather development	Chen et al. (2024)
MEGF11	Feather development	Twumasi et al. (2024)
MSRA	Altitude adaptation	Jeong et al. (2018), Gheyas et al. (2021)
AKT3	Altitude adaptation	Buroker et al. (2012), Qi et al. (2019)
DOCK11	Feather development	Crates et al. (2024)
SMARCA1	Craniofacial development	Boer et al. (2021)
GAB3	Craniofacial development	Boer et al. (2021)
ADGRD1	Altitude adaptation	Nan et al. (2023)
NPHP4	Vision	Borges et al. (2019)
ALDH7A1	Beak development	Bai et al. (2014)
ADAMTS19	Metabolism	Zhang et al. (2022)
VLDLR	Lipid protein	Wang et al. (2011)
ERCC8	Altitude adaptation	Yan et al. (2022)
CTIF	Beak development	Lawson and Petren (2017)

## 4 Discussion

Understanding the genetic and morphological basis of local adaptation is critical for predicting how species respond to environmental change. Alpine species face unique conservation challenges due to their extreme environments and thus are a high priority for studies of adaptive capacity. In this study, we investigated morphological and genetic differences between two populations of an alpine specialist songbird located in distinct parts of its environmental range. Consistent with our predictions, we found wings were shorter and feathers were denser in the colder environment. However, in contrast to our prediction, beak depth and width were larger in the colder environment, suggesting other factors like diet may place contrasting selective pressures on this ecologically important trait. We also identified numerous genes with known links to limb, facial, and feather development that potentially underlie variation in wing, bill, and feather microstructure. Further F_ST_ outlier analyses revealed numerous significant differences between cold and warm populations at SNPs located within genes with clear links to altitudinal adaptation. Together, our results provide important insights into the morphological traits and associated genomic regions associated with microgeographic variation in a high‐altitude songbird species.

### 4.1 Trait Differences Between Warm and Cold Habitats

Ecogeographic rules, such as Allen's and Bergmann's rules, are useful for predicting broad geographic trends in morphology in response to environmental conditions, but observed patterns do not always align with expectations. In our study, we found that wing chord was smaller in the colder environment, suggesting that this morphological trait may play a role in thermoregulation, consistent with Allen's rule. Heat dissipation from the wings occurs across the vascularized brachial regions, primarily during flight when these areas are exposed to air flow (Lewden et al. 2023; Ward et al. 1999). Wing bone length reflects the extent of vascularized areas on the wings and has been shown to be longer in warmer environments across a broad sample of passerine species (Weeks et al. 2025). However, studies of other alpine bird species have found the opposite pattern: That birds from high‐elevation sites have longer wings than those from low‐elevation sites (Bears et al. 2008; Ceresa et al. 2024). In these cases, researchers have suggested that the demand for more energetically efficient flight at high elevations may outweigh thermoregulatory selective pressures, demonstrating the often contrasting selective pressures on ecologically important traits. Although outside of the scope of the current work, additional wing measurements could be collected that would allow for a more thorough investigation into the components of flight morphology. It is possible that migratory behavior is influencing wing length as previous work has shown that both within (Egbert and Belthoff 2003; Grilli et al. 2017) and between (Lockwood et al. 1998) species those who migrate further have longer wings. To date, no formal study has been conducted on the migratory patterns of the Sierra Nevada Gray‐crowned subspecies and so future work could investigate the links between wing morphology and movement patterns. Overall, our morphological analyses support the idea that thermoregulatory demands may play an important role in shaping wing morphology in the Sierra Nevada Gray‐crowned Rosy‐Finch, potentially outweighing the benefits of flight efficiency in this high‐elevation, cold‐adapted species but not excluding the potentially parallel pressure of migratory distance.

Contrary to our predictions based on Allen's rule, beak depth and width were larger in the colder environment. Although temperature is often a key environmental variable exerting selective pressure on alpine taxa, variation in beak morphology between our populations may instead reflect divergent selective pressures associated with foraging ecology, a well‐documented driver of beak divergence in birds (Grant et al. 1976; Lamichhaney et al. 2018). In our study system, the Piute Pass population likely has access to more aquatic insects due to the higher availability of aquatic habitats proximate to Rosy‐Finch breeding sites in the Sierra Nevada (Epanchin et al. 2010; Rundel and Millar 2016). For example, Epanchin et al. (2010) found that, when abundant, mayflies can comprise up to 38% of the Sierra Nevada Gray‐Crowned Rosy‐Finch diet. In contrast, the White Mountain population may rely more heavily on insects deposited in snowfields, a behavior that could favor a more robust, conical bill for extracting frozen prey from the snow surface. Work is currently underway to investigate the dietary differences between these two populations of Rosy‐Finch (Tim Brown, personal correspondence), and future work should explore the selective pressures experienced by this species. The other aspect of beak morphology assessed, nare length, was significantly larger in the warmer environment. If nare is playing a role in thermoregulation, these results would be consistent with larger nares allowing for more heat to be lost. Additionally, smaller nares in the colder environment would potentially prevent snow from entering the nasal passages while foraging on snow. Overall, our results on beak morphology emphasize the complexity of identifying drivers of local adaptation, especially in alpine systems where extreme environments, food scarcity, and variation in migration strategies can create conflicting selection pressures.

Our comparison of body size did not have significant results, suggesting that Bergmann's rule is not contributing to morphological differences in this subspecies. He et al. (2023) found that there was significant variation in both strength and direction of correlation between latitude and body size within and between taxa. With our results, this could imply that Sierra Nevada Gray‐crowned Rosy‐Finch are an exception to the rule. In another meta‐analysis, it was found that sedentary bird species were more likely to conform to Bergmann's rule than migratory ones and suggest that winter temperatures may exert a higher pressure than breeding temperatures (Meiri and Dayan 2003). The Sierra Nevada Gray‐crowned Rosy‐Finch is a short‐distance, altitudinal migrant. It is possible that both White Mountain and Piute Pass populations move to similar areas or climates during the winter season, thus decreasing the divergent selection pressure that would drive differences in Bermann's rule.

Of our feather comparisons, we identified significant differences between cold and warm sites for only one trait: plumulaceous node density. In line with our predictions, plumulaceous node density was higher in the colder, high elevation White Mountain population. Higher node density is known to improve air trapping ability, thus trapping warmth against the body surface (King and McLelland 1984). Consistent with our results, a study on sparrows found that higher elevation alpine forms had higher node density relative to lowland forms (Lei et al. 2002). A further multispecies comparison found that species wintering in cold and windy conditions had downy feathers with higher node densities (D'alba et al. 2017). These interspecific trends are mirrored in our results, suggesting that the White Mountain population has feather adaptations consistent with an increased need for heat retention. The lack of significant results for other feather traits may be explained by the amount of variation in the measurements as well as the sample sizes being small, resulting in an underpowered comparison. An alternative cause for the non‐significant results is that alternative selection pressures not considered in the present work are driving morphology of these traits. This is somewhat supported by the overall low explanatory power of our models used in this study. Additional work on this aspect of local adaptation would benefit from investigating the influences of alternative environmental traits and utilizing a more controlled setting to evaluate the functionality of intraspecific differences in feather morphology.

### 4.2 Genome‐Wide Analysis

We used GWAS to identify SNPs within genes involved in traits that differ between warm‐ and cold‐adapted populations described previously: beak width and depth, nare length, wing cord length, and pennaceous barbule density. Because ours is one of the first studies to investigate the genetic basis of feather microstructure variation, we also report on the genes associated with other measured feather traits, even though high variance in these traits prevented our ability to robustly investigate differences between populations occupying cold and warm environments.

Our literature review of GWAS results identified genes with diverse associations. For wing chord, we identified limb and bone development, heat response, and vascularization. Notably, the genes *GUCA1C* and *NIPBL* have been linked to limb development in ducks and zebrafish https://www.zotero.org/google‐docs/?2z2bux (G. Li et al. 2020; Mohammadi 2024; Muto et al. 2014) and may play a similar developmental role in songbirds. We also detected the genes *PGLYRP2* and *PACRG*, which have been shown to be upregulated in chickens experiencing heat stress (Kim et al. 2021; Tian et al. 2020). Together, the limb development and heat response genes we identified as important further support the potential connection between wing length and thermoregulation suggested by our morphological finding of shorter wings in the colder environment. Although GWAS results do not establish a direct functional link to specific traits (Tam et al. 2019), our findings suggest that future research should investigate the potential role of thermoregulatory selective pressures on genes involved in wing development.

When focusing on genes associated with beak morphology, BSLMM found more SNPs than LMM and identified several genes potentially associated with facial structure development and climate adaptation. One gene associated with beak depth was *XIAP*. Duplications of this gene have been found to result in facial dysmorphism in humans, including the underdevelopment of cheekbones and protruding jaws (Di Benedetto et al. 2014). Although a link with beak development in birds has not yet been formally established, this could be an area for further research. Another gene identified for beak width was *MINDY2*, which belongs to a group of deubiquitinating enzymes. These genes are related to the regulation of the Wnt pathway (Park et al. 2020), a pathway that is well established as important for beak development (Geetha‐Loganathan et al. 2009). Additional genes such as *RASL11A, TINAG*, and *PLPP3* have been directly linked to beak development in avian species (Abernathy 2021; Bai et al. 2018). Two genes associated with beak depth in our study (*LRP1B* and *UTRN*) were shown to be associated with breeding temperature in a study that used a GWAS approach to identify loci associated with climate variables related to morphology in hermit thrush (N. Adams et al. 2025). Although the beak morphology of our species did not entirely fall in line with predictions based around their thermoregulatory role, the elucidation of genes indicating a relationship between thermoregulation and beak morphology suggests that beaks still play a thermoregulatory role in this species.

Our GWAS on feather traits was, to the best of our knowledge, the first genome‐wide association study of feather microstructural traits. Identifying the genetic basis of these fine‐scale traits opens new avenues for studying how feather structure evolves in response to environmental pressures and functional demands. One of the genes associated with pennaceous barbule length in our study, *HBE1*, has also been identified in previous research on feather development. Using RNA sequencing, Limber et al. (2024) found that *HBE1*, a hemoglobin gene (Mao et al. 2023), was highly expressed in feather pulp, the central tissue of the developing feather germ. Additional genes associated with feather traits in our study have been identified as being related to plumage coloration in birds, including *EFNA2, NRXN1*, and *HERC4* (Shakya 2020; L. Sun et al. 2020; X. Zhang et al. 2023). Many other genes identified as being associated with feather traits in our work have not been shown to be associated with feather development or morphology in the literature. Feather morphology remains understudied in the context of local adaptation, and the genetic architecture of these microstructural traits is still poorly understood. Our study offers a foundation for deeper investigation. Expanding this work to include comparative genomic studies across populations or species with more strongly differentiated feather morphology could also help link genotype to phenotype and provide insight into the adaptive significance of feather microstructure.

It is important to note that, in this system, we are limited in our ability to assess the role of phenotypic plasticity in driving trait differences. Bringing individuals together from both populations into a common aviary, as was done in Bears et al. (2008), would allow for better examination of these effects. It could also be argued that showing relatively high F_ST_ values for GWAS loci might suggest that the morphological differentiation is due to divergence rather than phenotypic plasticity. Our original intent with the F_ST_ outlier tests was to determine if any of the genes identified in the GWAS had variants that diverged significantly between the cold and warm environment populations. However, when comparing our F_ST_ results with SNPs identified in the GWAS, we found no overlap between the two sets. Although this could suggest that phenotypic plasticity is primarily driving the trait divergence, one must also consider that this result is consistent with known limitations of F_ST_‐based outlier scans, which are biased toward detecting loci under strong divergent selection and with large effect sizes (Hoban et al. 2016). F_ST_ measures allele frequency differences between populations, and loci with large phenotypic effects are more likely to experience strong selection and exhibit pronounced divergence. In contrast, highly polygenic traits are shaped by small‐effect variants that shift subtly in frequency—patterns that often fall below the detection threshold of F_ST_ scans. Our findings underscore the value of combining F_ST_ outlier analyses with GWAS approaches: while the former is designed to pinpoint loci under strong selection, the latter is better suited for uncovering the complex genetic architecture of polygenic traits. Together, these methods can generate a more complete understanding of the genetic basis for local adaptation.

While we did not find overlap between the F_ST_ and the GWAS results, our F_ST_ analysis highlighted numerous genes involved in hypoxia response, altitude adaptation, and cold adaptation. These results are consistent with our hypothesis that elevation and associated climatic variables are applying divergent selection pressures in this species. Of these genes, *NOX4* was found to be involved in mediating hypoxia‐inducible transcription factors, a key component of the cellular response to hypoxia (Diebold et al. 2010). Similarly, *MAML2* and *SUPT20H* were found to be associated with altitude adaptation in deer mice (Schweizer et al. 2021), and *ADGRD1* was associated with altitude adaptation in Tibetan chickens (Nan et al. 2023). Multiple studies identified *PPP1R1C* as being related to cold adaptation in chickens (Fedorova et al. 2022; Romanov et al. 2023). These are all traits that would lend themselves to survival in alpine environments, and divergent selection is likely a product of varying selection pressures between the higher and colder White Mountain location and the warmer and lower Piute Pass location.

Our F_ST_ analysis also identified several genes that might be involved with plumage color differences in Rosy‐Finches. Another gene that showed divergence between these two populations of finch was *PHIP*, which has been shown to be related to differentiation in plumage coloration in House Finch (Balenger et al. 2015). Interestingly, when comparing candidate outlier genes from this work with studies identifying genes involved with the notable plumage coloration differences across the broader rosy‐finch species complex, there were some overlapping genes. In a GWAS on crown coloration, Funk et al. (2023) identified *NDUFAF2*, a gene that was also significant in this work. For body plumage coloration, our results overlap with those of Funk et al. (2023) for the genes *KCNV2*, *PUM3*, and *VLDLR*. Although it was not a component of the current work, other studies have shown that plumage coloration is involved in avian thermoregulation, with darker plumage putatively facilitating heat absorption (Medina et al. 2018; Rogalla et al. 2022), and can show adaptation to local environment (Romano et al. 2019; Sandoval and Barrantes 2019; Sirkiä et al. 2010). This provides an important avenue for future work with this species investigating the differences in plumage coloration between populations.

An additional result of our F_ST_ analysis was the identification of a gene, *CTIF*, that has been previously identified as being a climate‐adapted gene in a landscape genomic study on the closely related Brown‐capped Rosy‐Finch (DeSaix et al. 2022). Interestingly, this gene was also identified through a contrasting approach using a mixed linear model on beak shape for two species of Darwin's finches, *Geospiza fortis* and *G. scandens* (Lawson and Petren 2017). The authors of this paper summarize their results as indicating that inter and intraspecies beak shape variation is a result of a small suite of traits evolving in concert, corresponding to many genes. It is possible that we did not identify *CTIF* genes in our GWAS method because of low power due to smaller variation in the beak traits measured, a result of using continuous measurements between two populations versus categorical comparisons between different species as in Lawson and Petren (2017). Because of the large number of SNPs obtained for our two populations, our F_ST_ analysis likely had higher power and thus detected this gene, in addition to the fact that it had a high F_ST_ of 0.263 (averaged across the 5 SNPs associated with this trait). The overlap of these genes in the two studies described, as well as ours, makes it a strong candidate as a genetic component of adaptation to environment, potentially through the mechanism of beak morphology.

An important outcome of local adaptation is the maintenance of ecologically important genetic variation (Savolainen et al. 2013). Conserving genetic variation prevents loss of adaptive potential and can underlie rapid adaptive responses to environmental change (Forester et al. 2023). We integrated phenotypic measurements, genome‐wide association studies, and F_ST_ analyses to understand the morphological and genetic basis of local adaptation of an alpine bird species, the Sierra Gray‐crowned Rosy‐Finch. When comparing traits suspected to be involved in thermoregulation for this species, we found that morphological variation across populations only partially aligned with ecogeographic expectations. Wing chord was shorter in the colder environment, consistent with Allen's rule and likely reflecting thermoregulatory adaptation. However, beak depth and width were larger in colder environments, contrary to predictions, suggesting additional selective pressures such as diet specialization may be playing a role. Comparisons of feather microstructure further showed thermoregulatory adaptation, with higher plumulaceous node density in the high‐elevation population likely enhancing insulation. When coupled with the results of our GWAS and F_ST_ outlier scans, we gained a clearer picture of traits and genes important to adaptation in alpine birds. Importantly, we found that many of the genes underlying wing length, beak depth, and feather microstructure had links to both development of similar traits in other organisms as well as thermoregulation. Our genomic and morphological results provide evidence that this alpine species is locally adapted to temperature variation.

For those traits that are involved in thermoregulation, we can hypothesize about the implication of the intraspecific differences in light of future climate change. Recent work has shown that, for populations whose morphology and genetics misalign with the environment as it changes due to anthropogenic pressures, there is a decrease in fitness (Pelletier and Coltman 2018; Rodriguez et al. 2025). As climate change drives increasing variability in temperature, understanding both simple and complex genetic architectures underlying local adaptation is essential for predicting species' responses to global change. Although we cannot directly assess fitness consequences in this species, this work enhances our understanding of the morphological traits and genetic mechanisms underlying local adaptation in an alpine species and lays a groundwork for future investigation. The resulting insights will be used to inform the delineation of conservation units and support management strategies that seek to preserve the evolutionary processes essential for long‐term persistence of alpine organisms in a changing world (Allendorf et al. 2010; Flanagan et al. 2018; Shafer et al. 2015).

## Author Contributions

**Erica C. N. Robertson:** conceptualization (supporting), formal analysis (lead), methodology (lead), writing – original draft (equal). **Timothy M. Brown:** conceptualization (supporting), methodology (supporting), writing – review and editing (equal). **Sophie Deitch:** methodology (supporting), writing – original draft (supporting). **Christine M. Bossu:** formal analysis (supporting), methodology (supporting), writing – original draft (supporting). **Erika S. Zavaleta:** conceptualization (supporting), funding acquisition (equal), methodology (supporting), writing – review and editing (equal). **Mevin B. Hooten:** funding acquisition (equal), writing – review and editing (equal). **Kristen C. Ruegg:** conceptualization (supporting), funding acquisition (equal), methodology (supporting), supervision (equal), writing – review and editing (equal).

## Funding

This work was supported by the National Science Foundation Grant No. 2222524, 2222525, and 2222526.

## Disclosure

Benefit‐sharing statement: Benefits from this research accrue from the sharing of our data and results on public databases as described above.

## Conflicts of Interest

The authors declare no conflicts of interest.

## Supporting information


**Data S1:** ece372962‐sup‐0001‐Supinfo.zip.

## Data Availability

Raw sequence data have been deposited in the NCBI Sequence Read Archive under BioProject accession PRJNA1297057. Statistical analysis, GWAS and F_ST_ results are available at Dryad (DOI: 10.5061/dryad.kwh70rzhs). Code and scripts used for processing and analysis of sequence data are available at https://github.com/eriqande/mega‐non‐model‐wgs‐snakeflow/. All other scripts as well as sample metadata are available on GitHub at https://github.com/ecnrobertson/GCRF_Pub.
