# Correction to ‘The Genetic and Morphological Basis of Local Adaptation to Elevational Extremes in an Alpine Finch’

**DOI:** 10.1002/ece3.73162

**Published:** 2026-02-23

Robertson et al. 2026. ‘The Genetic and Morphological Basis of Local Adaptation to Elevational Extremes in an Alpine Finch.’ *Ecology and Evolution* 16, no. 2: e72962. https://doi.org/10.1002/ece3.72962.

The author order in the original publication of this article was incorrect. Erika S. Zavaleta was originally listed as the third‐to‐last author; however, she should be listed as the second‐to‐last author. Additionally, the name ‘Christine M. Bossu’ should be corrected to ‘Christen M. Bossu’. The correct author list is as follows:

Erica C. N. Robertson, Timothy M. Brown, Sophie Deitch, Christen M. Bossu, Mevin B. Hooten, Erika S. Zavaleta, Kristen C. Ruegg.

The online version of this article has been corrected accordingly.

We apologize for these errors.

