# Supplementary material for: The Genetic and Morphological Basis of Local Adaptation to Elevational Extremes in an Alpine Finch
Source: Ecol Evol. 2026 Jan 29;16(2):e72962. doi: 10.1002/ece3.72962 (PMC12853319; doi:10.1002/ece3.72962)

**Supplemental Information for:**

The genetic and morphological basis of local adaptation to elevational extremes in an alpine finch

Robertson, Erica C.N.^1^, Brown, Timothy^2^, Deitch, Sophie^1^, Bossu, Christine M.^1^, Zavaleta, Erika S.^2^, Hooten, Mevin B.^3^, Ruegg, Kristen C.^1^

**Supplemental Methodology**

*Extracting temperature data from AdaptWest, 2022.*

The two sampling locations, identified by GPS coordinates collected during fieldwork, were each buffered by 1 km to account for the spatial heterogeneity experienced by the birds. Monthly climate rasters from AdaptWest were filtered to include only June, July, and August, corresponding to the Gray-crowned Rosy-Finch breeding season. For each site, raster layers representing mean, minimum, and maximum monthly temperature were extracted within the 1 km buffer, and pixel values were averaged. The resulting mean, minimum, and maximum temperature values were then summarized for each site. The average values were also plotted across time (Sup
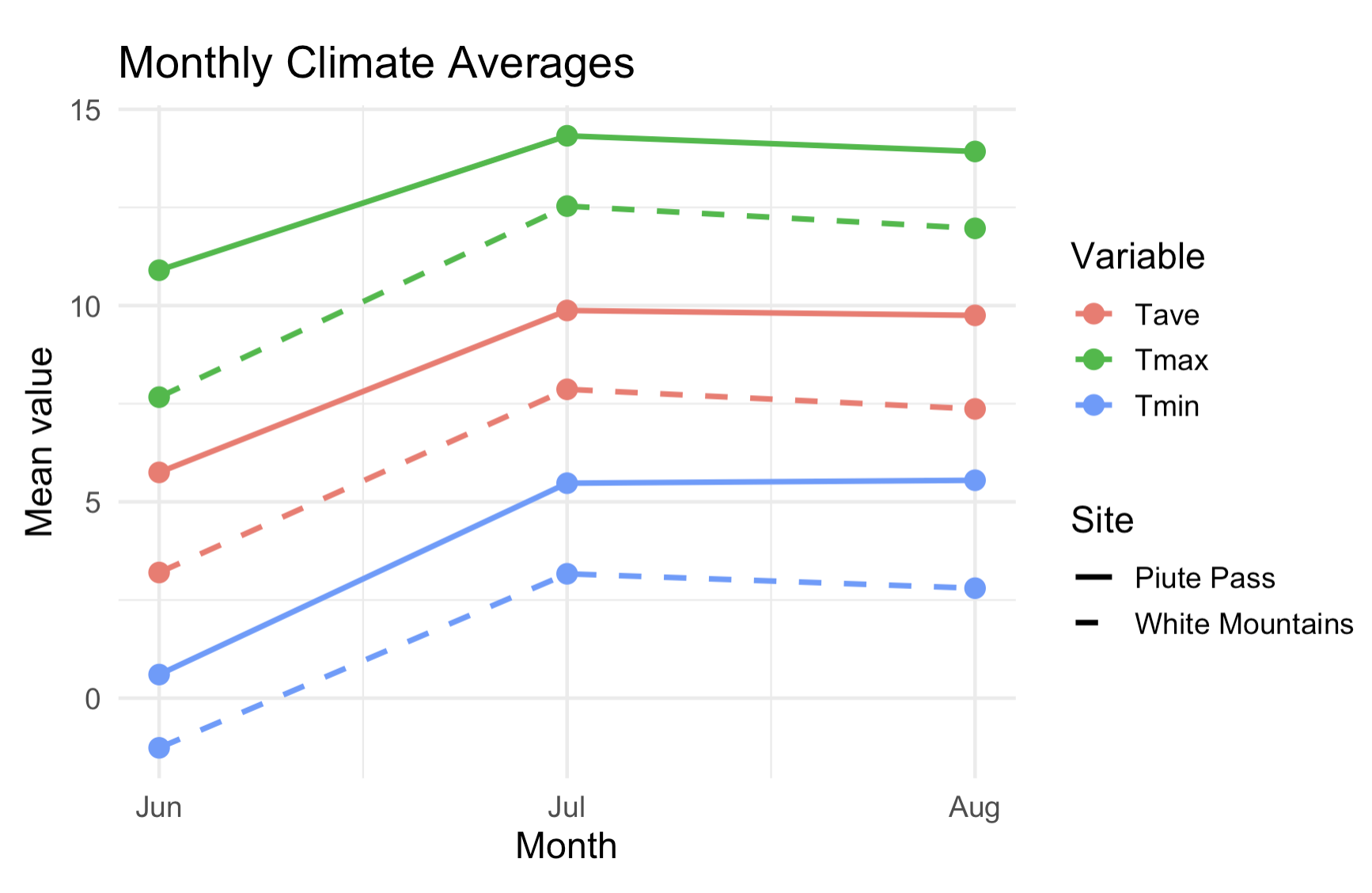


Fig 1).

Sup Fig 1. Average values within a 1km buffer around sample sites for Tave, Tmax, and Tmin of the breeding season plotted against month.

*Sexing and Aging of Birds*

Aging followed standard guidelines outlined in the *Pyle Identification Guide to North American Birds* (Pyle 1997). Age was determined primarily by examination of skull pneumatization and degree of cranial ossification, supplemented by plumage characteristics (extent of molt limits, feather wear, and coloration). When available, known-age individuals from previous banding or recapture records were also included. All assessments were conducted by trained banders using consistent criteria across field seasons. A full breakdown of individuals by age class and sex is provided in Sup Table 1.

*Variation around morphological data collected*

Not all individuals were measured for the full suite of morphological traits. In some cases, birds exhibited signs of acute handling stress (e.g., wincing of the eyes, open-beak panting, or rapid breathing), and were released before all measurements could be completed to minimize risk. In other instances, data collection was interrupted by rapidly changing field conditions, such as approaching thunderstorms or hail events, which required immediate cessation of banding activities for personnel safety. As a result, sample sizes differ slightly among traits, reflecting our decision to prioritize bird welfare and field safety over completeness of measurements.

*Generating PC axis representing size*

To further validate our choice of tarsus as a proxy for body size, we performed a principal component analysis on allometric traits to compare a PC axis representing overall body size with tarsus. We first removed all individuals that were missing information for any of the following traits: wing chord, tarsus, tail length, beak depth, beak width, and beak length. This left us with 136 individuals with complete data. We centered and scaled the variables and performed a principal component analysis on the resulting data. The first axis, PC1, explained 34.3% of the variance while PC2 explained 28.7%. We found the following loadings for PC1 and PC2:

|  | wing chord | tarsus | tail length | beak depth | beak width | beak length |
| --- | --- | --- | --- | --- | --- | --- |
| PC1 | 0.5900 | 0.5358 | 0.5358 | 0.2094 | 0.1290 | 0.0172 |
| PC2 | -0.1654 | -0.0829 | -0.1260 | 0.6134 | 0.5789 | 0.4881 |

Because of the highly positive loadings on PC1 and the high percent of variance explained, we used this as a proxy for body size. We found that PC1_size was highly correlated with tarsus (0.77) so we proceeded with tarsus as our proxy for body size.

**Morphological data and analysis**

*Summary of morphological data*

Sup Table 1. Summary of sample sizes, distribution of sexes and ages for each site.

| **Piute Pass n=72** | | **White Mountains n=98** | |
| --- | --- | --- | --- |
| **Sex** | **Age** | **Sex** | **Age** |
| F:25 | SY:30 | F:42 | SY:55 |
| M:35 | ASY:30 | M:35 | ASY:22 |
| UNK:12 | HY:12 | UNK:21 | HY:21 |

Sup Table 2. Summary of trait values for Piute Pass.

| **Trait** | **Mean** | **SD** | **N** | **Range** |
| --- | --- | --- | --- | --- |
| Wing Chord | 100.47 | 3.808 | 72 | 92-110 |
| Tarsus | 20.001 | .729 | 72 | 18.1-22.3 |
| Nare Length | 2.186 | 0.4362 | 57 | 1.45-3.2 |
| Beak Depth | 6.505 | 0.544 | 61 | 4.74-7.79 |
| Beak Width | 5.837 | 0.5310 | 61 | 4.5-7.07 |
| Beak Length | 11.504 | 0.5686 | 61 | 9.4-12.87 |
| Pen Barbule Length | 0.3795 | 0.0593 | 54 | 0.29-0.61 |
| Pen Barbule Density | 19.276 | 3.1449 | 54 | 13.25-24.67 |
| Plum Barbule Length | 0.8243 | 0.0902 | 54 | 0.64-1.05 |
| Plum Barbule Density | 28.353 | 2.6963 | 54 | 21-33.33 |
| Plum Barbule Node Density | 6.997 | 0.5748 | 54 | 5.33-8 |

Sup Table 3. Summary of trait values for White Mountain.

| **Trait** | **Mean** | **SD** | **N** | **Range** |
| --- | --- | --- | --- | --- |
| Wing Chord | 98.44 | 3.435 | 95 | 92-106 |
| Tarsus | 19.9 | .572 | 95 | 18.4-21.7 |
| Nare Length | 1.87 | 0.327 | 93 | 1.14-2.9 |
| Beak Depth | 6.569 | 0.4757 | 95 | 5.56-7.97 |
| Beak Width | 6.0202 | 0.4743 | 95 | 5-7 |
| Beak Length | 11.2 | 0.6844 | 95 | 9.49-12.88 |
| Pen Barbule Length | 0.3631 | 0.05541 | 85 | 0.24-0.52 |
| Pen Barbule Density | 19.8313 | 2.6285 | 85 | 13.67-25.33 |
| Plum Barbule Length | 0.79822 | 0.1011 | 85 | 0.6-1.11 |
| Plum Barbule Density | 28.38039 | 3.0564 | 85 | 20.33-35 |
| Plum Barbule Node Density | 7.2745 | 0.6865 | 85 | 5.33-9.33 |

**Morphological Statistical Analysis Model Details**

*Univariate Model Summary Details*

We evaluated a suite of candidate linear models for each morphological trait to assess the effects of body size (tarsus length), age, sex, and site. All models were fit using ordinary least squares in R. The following fixed-effect combinations were tested:

| **Model ID** | **Fixed Effects Included** |
| --- | --- |
| 1 | tarsus |
| 2 | age |
| 3 | sex |
| 4 | site_code |
| 5 | tarsus + age |
| 6 | tarsus + sex |
| 7 | tarsus + site_code |
| 8 | age + sex |
| 9 | age + site_code |
| 10 | sex + site_code |
| 11 | tarsus × age |
| 12 | tarsus × sex |
| 13 | tarsus × site_code |
| 14 | age × sex |
| 15 | age × site_code |
| 16 | sex × site_code |
| 17 | tarsus + age + sex |
| 18 | tarsus + age + site_code |
| 19 | tarsus + sex + site_code |
| 20 | age + sex + site_code |
| 21 | tarsus × age × sex |
| 22 | tarsus × age × site_code |
| 23 | tarsus × sex × site_code |
| 24 | age × sex × site_code |

Sup Table 4. Top three models tested and resulting AIC information for each of those models for each trait.

| **Formula** | **K** | **AICc** | **Delta_AICc** | **AICcWt** | **Cum.Wt** |
| --- | --- | --- | --- | --- | --- |
| Beak Depth | | | | | |
| tarsus + age + site_code | 6 | 189.95893 | 0 | 0.221979855 | 0.221979855 |
| tarsus + sex + site_code | 6 | 190.2100988 | 0.251168766 | 0.195782089 | 0.417761944 |
| tarsus * sex * site_code | 12 | 190.6927073 | 0.73377728 | 0.153806911 | 0.571568855 |
| Beak Width | | | | | |
| tarsus + sex + site_code | 6 | 214.6032667 | 0 | 0.281768018 | 0.281768018 |
| tarsus * sex * site_code | 12 | 214.7332994 | 0.130032724 | 0.264031322 | 0.54579934 |
| tarsus + age + sex + site_code | 7 | 216.5458443 | 1.942577589 | 0.106675904 | 0.652475245 |
| Beak Length | | | | | |
| sex | 4 | 202.8584822 | 0 | 0.210260541 | 0.210260541 |
| age | 4 | 204.0543127 | 1.195830512 | 0.115634248 | 0.325894789 |
| tarsus + sex | 5 | 204.2756416 | 1.41715939 | 0.103520301 | 0.42941509 |
| Nare Length | | | | | |
| tarsus + sex + site_code | 6 | 124.9657502 | 0 | 0.350894556 | 0.350894556 |
| tarsus + age + sex + site_code | 7 | 125.2809829 | 0.315232657 | 0.299726215 | 0.650620771 |
| tarsus + age + site_code | 6 | 127.3203308 | 2.354580554 | 0.108115002 | 0.758735773 |
| Wing Chord | | | | | |
| tarsus + age + sex + site_code | 7 | 735.5245212 | 0 | 0.83590162 | 0.83590162 |
| tarsus + sex + site_code | 6 | 738.8588978 | 3.334376595 | 0.15779909 | 0.9937007 |
| tarsus + age + sex | 6 | 745.5227036 | 9.998182374 | 0.00563738 | 0.99933809 |
| Pennaceous Barbule Length | | | | | |
| sex | 4 | -445.9251986 | 0 | 0.265387099 | 0.265387099 |
| sex + site_code | 5 | -445.551068 | 0.374130643 | 0.220109288 | 0.485496387 |
| age * sex | 6 | -444.4421004 | 1.483098222 | 0.126423881 | 0.611920268 |
| Pennaceous Barbule Density | | | | | |
| tarsus + age | 5 | 616.5544216 | 0 | 0.248150548 | 0.248150548 |
| tarsus + sex | 5 | 616.9388184 | 0.384396855 | 0.204759718 | 0.452910266 |
| tarsus + age + site_code | 6 | 617.9156512 | 1.361229618 | 0.125640016 | 0.578550283 |
| Plumulaceous Barbule Length | | | | | |
| age + site_code | 5 | -267.5849449 | 0 | 0.276570322 | 0.276570322 |
| age | 4 | -266.5360443 | 1.048900587 | 0.163696618 | 0.44026694 |
| age * site_code | 7 | -266.5107537 | 1.074191168 | 0.16163966 | 0.6019066 |
| Plumulaceous Barbule Density | | | | | |
| tarsus + age | 5 | 672.4422346 | 0 | 0.30687339 | 0.30687339 |
| tarsus + age + sex | 6 | 673.1769641 | 0.734729498 | 0.212527311 | 0.519400701 |
| tarsus + sex | 5 | 673.6425194 | 1.20028475 | 0.168391711 | 0.687792412 |
| Plumulaceous Barbule Node Density | | | | | |
| tarsus + age + site_code | 6 | 229.2717093 | 0 | 0.4507249 | 0.4507249 |
| age + site_code | 5 | 231.33502 | 2.063310625 | 0.160645787 | 0.611370687 |
| tarsus + age + sex + site_code | 7 | 231.48286 | 2.211150657 | 0.149199129 | 0.760569816 |

Sup Table 5. Summary of model ran and the model results for the top model for each trait. Asterisk (*) notes which traits are considered significantly different between sites.

| **Formula** | **r.squared** | **adj.r.squared** | **p.value** |
| --- | --- | --- | --- |
| Beak Depth * | | | |
| tarsus + age + site_code | 0.26910403 | 0.24961347 | 1.30E-09 |
| Beak Width * | | | |
| tarsus + sex + site_code | 0.15702313 | 0.13454374 | 3.49E-05 |
| Beak Length | | | |
| sex | 0.52253017 | 0.51628873 | 2.75E-25 |
| Nare Length * | | | |
| tarsus + sex + site_code | 0.219043 | 0.19719804 | 3.50E-07 |
| Wing Chord * | | | |
| tarsus + age + sex + site_code | 0.6826689 | 0.6728139 | 2.35E-38 |
| Pennaceous Barbule Length | | | |
| sex | 0.31689028 | 0.30684455 | 5.56E-12 |
| Pennaceous Barbule Density | | | |
| tarsus + age | 0.3791724 | 0.36506268 | 1.25E-13 |
| Plumulaceous Barbule Length * | | | |
| age + site_code | 0.16133686 | 0.1426999 | 2.70E-05 |
| Plumulaceous Barbule Density | | | |
| tarsus + age | 0.09665926 | 0.07612878 | 0.00372514 |
| Plumulaceous Barbule Node Density | | | |
| tarsus + age | 0.09665926 | 0.07612878 | 0.00372514 |

Sup Table 6.A-J. Detailed summary for each of the trait models run. Sample size (n) is noted for each analysis. Significant site p-values are italicized. When site is present, 95% CI is also shown with the beta coefficient for site.

A. Beak Depth, n=155

| **term** | **estimate** | **std.error** | **statistic** | **p.value** |
| --- | --- | --- | --- | --- |
| (Intercept) | 3.37921794 | 1.20275672 | 2.80956065 | 0.00562258 |
| tarsus | 0.15757233 | 0.06065696 | 2.59776171 | 0.0103184 |
| age3 | -0.0419748 | 0.08464803 | -0.4958742 | 0.62070894 |
| age4 | -0.7813467 | 0.11173235 | -6.9930214 | 8.29E-11 |
| *site_codeWMTN* | *0.23301356* | *0.0764162* | *3.04926899* | *0.00271257* |
|  | *β = 0.46, 95% CI = 0.16–0.76* | | | |

B. Beak Width, n=155

| **term** | **estimate** | **std.error** | **statistic** | **p.value** |
| --- | --- | --- | --- | --- |
| (Intercept) | 3.13067772 | 1.26675195 | 2.47142127 | 0.01457617 |
| tarsus | 0.13913241 | 0.06376169 | 2.18206908 | 0.03066072 |
| sexM | -0.1290975 | 0.0855716 | -1.5086492 | 0.13349188 |
| sexUNK | -0.5671672 | 0.12422359 | -4.5656968 | 1.03E-05 |
| *site_codeWMTN* | *0.28128538* | *0.0818869* | *3.43504748* | *0.0007664* |
|  | *β = 0.55, 95% CI = 0.24–0.87* | | | |

C. Beak Length, n=156

| **term** | **estimate** | **std.error** | **statistic** | **p.value** |
| --- | --- | --- | --- | --- |
| (Intercept) | 11.5557576 | 0.05611459 | 205.931438 | 6.60E-189 |
| sexM | -0.0851779 | 0.07849068 | -1.0851971 | 0.27954128 |
| sexUNK | -1.4224242 | 0.11421569 | -12.453843 | 5.00E-25 |

D. Nare Length, n=148

| **term** | **estimate** | **std.error** | **statistic** | **p.value** |
| --- | --- | --- | --- | --- |
| (Intercept) | 0.00316755 | 0.92079149 | 0.00344003 | 0.99726005 |
| tarsus | 0.11403506 | 0.04632887 | 2.46142557 | 0.01502832 |
| sexM | -0.1216435 | 0.06971025 | -1.7449874 | 0.08313529 |
| sexUNK | -0.2391738 | 0.07966172 | -3.0023678 | 0.0031627 |
| *site_codeWMTN* | *-0.3084156* | *0.0611737* | *-5.0416373* | *1.38E-06* |
|  | *β = -0.76, 95% CI = -1.07-(-0.46)* | | | |

E. Wing Chord, n=167

| **term** | **estimate** | **std.error** | **statistic** | **p.value** |
| --- | --- | --- | --- | --- |
| (Intercept) | 67.5307604 | 5.49907378 | 12.2803881 | 6.99E-25 |
| tarsus | 1.47810264 | 0.27931635 | 5.29185855 | 3.91E-07 |
| age3 | 0.98013348 | 0.42161033 | 2.32473783 | 0.02133471 |
| age4 | 2.61989277 | 0.47482379 | 5.51761055 | 1.35E-07 |
| sexM | 5.18357951 | 0.39536383 | 13.1109099 | 3.49E-27 |
| *site_codeWMTN* | *-1.1884579* | *0.34055186* | *-3.4898001* | *0.0006237* |
|  | *β = -0.32, 95% CI = -0.50-(-0.14)* | | | |

F. Pennaceous Barbule Length, n=139

| **term** | **estimate** | **std.error** | **statistic** | **p.value** |
| --- | --- | --- | --- | --- |
| (Intercept) | 0.34640226 | 0.00621558 | 55.7312693 | 1.55E-95 |
| sexM | 0.01699774 | 0.00899133 | 1.89045812 | 0.06082431 |
| sexUNK | 0.0882362 | 0.0112384 | 7.85131195 | 1.10E-12 |

G. Pennaceous Barbule Density, n=136

| **term** | **estimate** | **std.error** | **statistic** | **p.value** |
| --- | --- | --- | --- | --- |
| (Intercept) | 23.2506487 | 6.11112099 | 3.80464545 | 0.00021631 |
| tarsus | -0.1510297 | 0.30918622 | -0.4884749 | 0.626024 |
| age3 | 0.47029958 | 0.47571193 | 0.98862262 | 0.32465654 |
| age4 | -4.24769 | 0.53314041 | -7.9673009 | 6.67E-13 |

H. Plumulaceous Barbule Length

| **term** | **estimate** | **std.error** | **statistic** | **p.value** |
| --- | --- | --- | --- | --- |
| (Intercept) | 0.8237826 | 0.01620735 | 50.8277177 | 6.72E-90 |
| age3 | -0.0349157 | 0.01789566 | -1.9510714 | 0.0531196 |
| age4 | 0.07217363 | 0.02106868 | 3.42563687 | 0.00081297 |
| site_codeWMTN | -0.0288163 | 0.01624784 | -1.773549 | 0.07839249 |
|  | β = -0.30, 95% CI = -0.62-0.34 | | | |

I. Plumulaceous Barbule Density

| **term** | **estimate** | **std.error** | **statistic** | **p.value** |
| --- | --- | --- | --- | --- |
| (Intercept) | 25.5415447 | 7.50508001 | 3.40323417 | 0.00088205 |
| tarsus | 0.1335034 | 0.37971223 | 0.351591 | 0.72570563 |
| age3 | 1.16460095 | 0.58422278 | 1.99341926 | 0.048277 |
| age4 | -1.3306565 | 0.65475081 | -2.0323098 | 0.04412831 |

J. Plumulaceous Barbule Node Density

| **term** | **estimate** | **std.error** | **statistic** | **p.value** |
| --- | --- | --- | --- | --- |
| (Intercept) | 9.17335111 | 1.46944391 | 6.24273647 | 5.54E-09 |
| tarsus | -0.1062565 | 0.07416009 | -1.432799 | 0.15429738 |
| age3 | 0.27870664 | 0.11659663 | 2.39034915 | 0.01825573 |
| age4 | -0.7579252 | 0.12906466 | -5.8724455 | 3.34E-08 |
| *site_codeWMTN* | *0.26874166* | *0.0987462* | *2.72153933* | *0.00738179* |
|  | β = 0.41, 95% CI = 0.11-0.70 | | | |

**Population Structure Results**


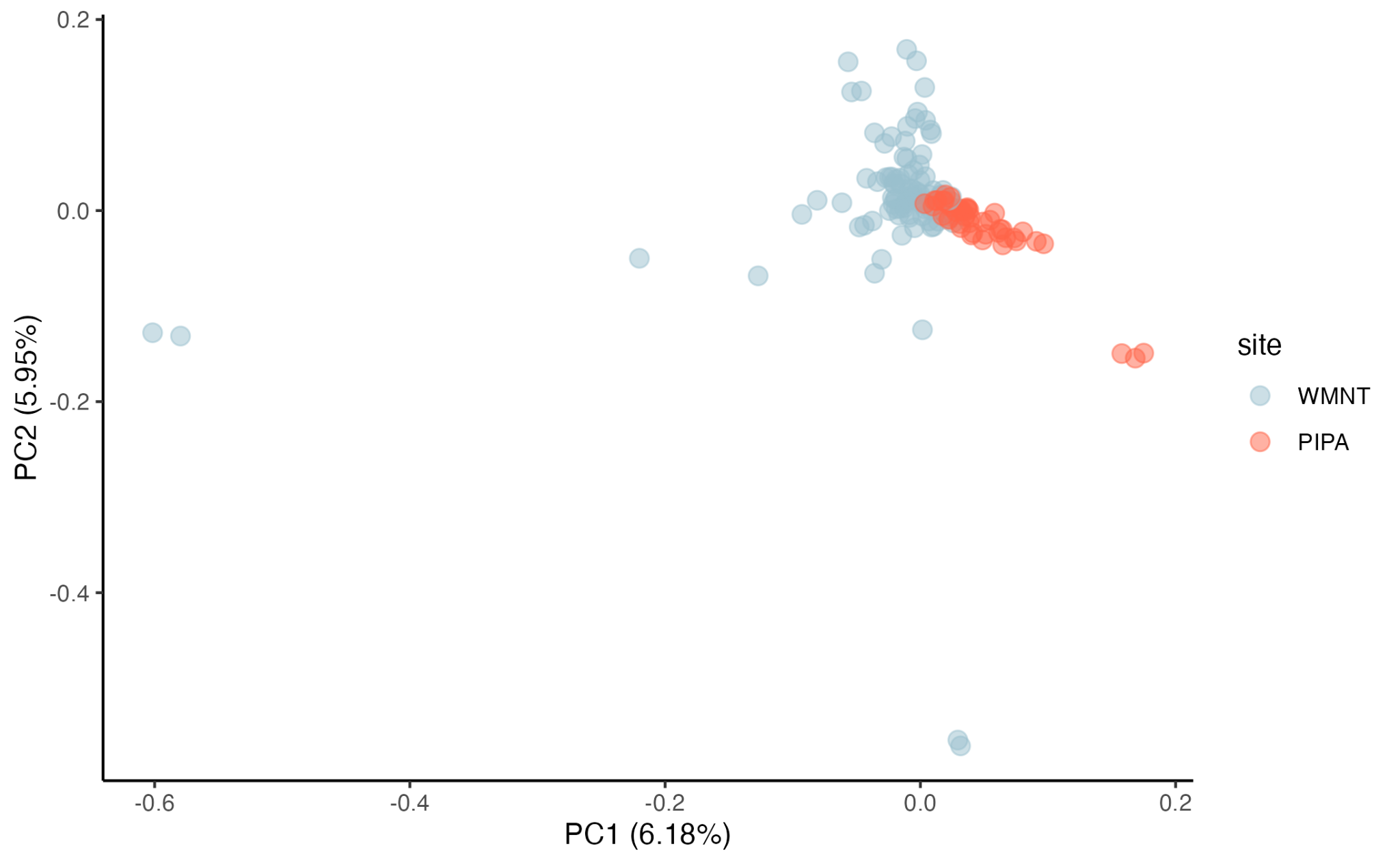


Sup Fig 2. PCA results showing a lack of population structure between the White Mountain (WMNT, blue) and Piute Pass (PIPA, red) populations. There are some outlier individuals but an ADMIXTURE analysis confirmed that 1 population is the most supported.

**Extended GWAS Results**

**
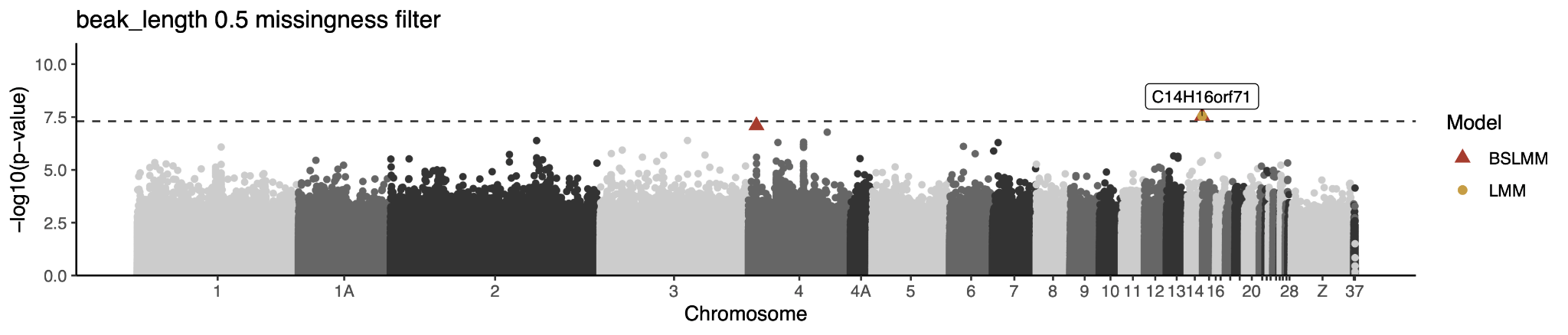
**

Sup Fig 3. Beak length GWAS results. Significant SNPs from the BSLMM analysis are overlaid onto the LMM results, plotted with their corresponding p-values and corresponding genes are noted if available.


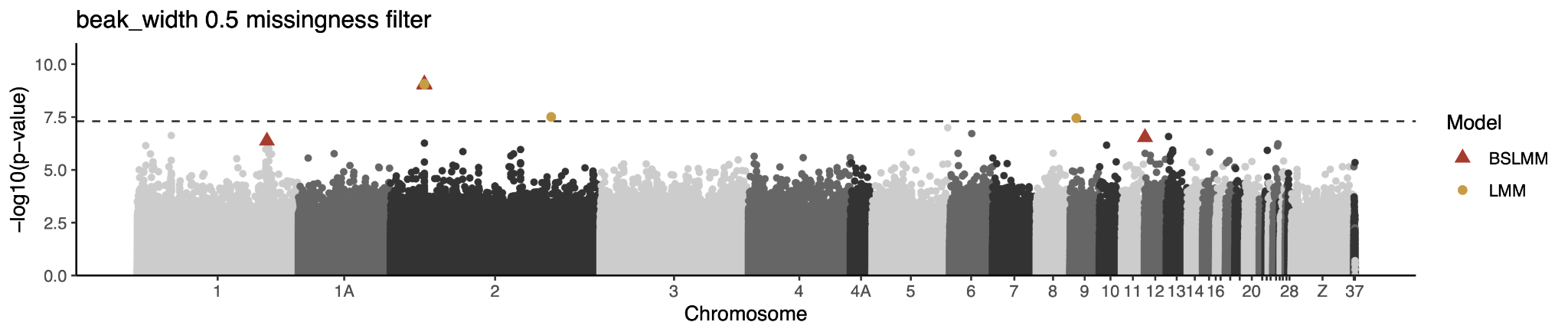


Sup Fig 4. Beak width GWAS results. Significant SNPs from the BSLMM analysis are overlaid onto the LMM results, plotted with their corresponding p-values and corresponding genes are noted if available.


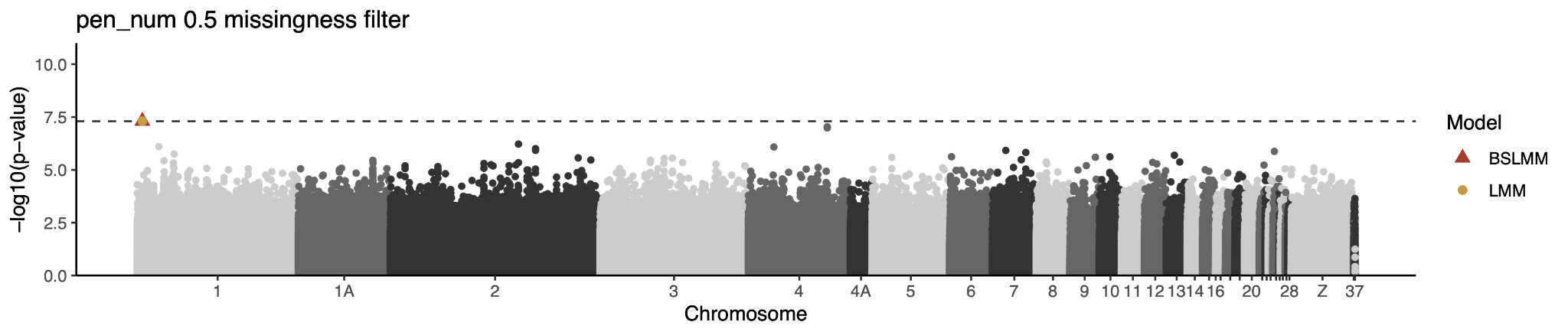


Sup Fig 5. Pennaceous barbule density GWAS results. Significant SNPs from the BSLMM analysis are overlaid onto the LMM results, plotted with their corresponding p-values and corresponding genes are noted if available.

**GWAS model evaluation**

Sup Table 7. λGC value for each ULMM run, based on p-values.

| Trait | λGC |
| --- | --- |
| Wing Chord | 1.0287 |
| Nare Length | 1.0316 |
| Beak Depth | 1.0129 |
| Beak Width | 1.0157 |
| Beak Length | 0.9684 |
| Pen Barbule Length | 1.0273 |
| Pen Barbule Density | 1.0232 |
| Plum Barbule Length | 0.9773 |
| Plum Barbule Density | 1.0009 |
| Plum Barbule Node Density | 1.0218 |

Sup Fig 6. PP plots for all traits: A) plumulaceous barbule density, B) plumulaceous barbule node density, C) pennaceous barbule density, D) pennaceous barbule length, E) plumulaceous barbule length, F) beak length, G) beak width, H) beak depth, I) nare length, J) wing chord.


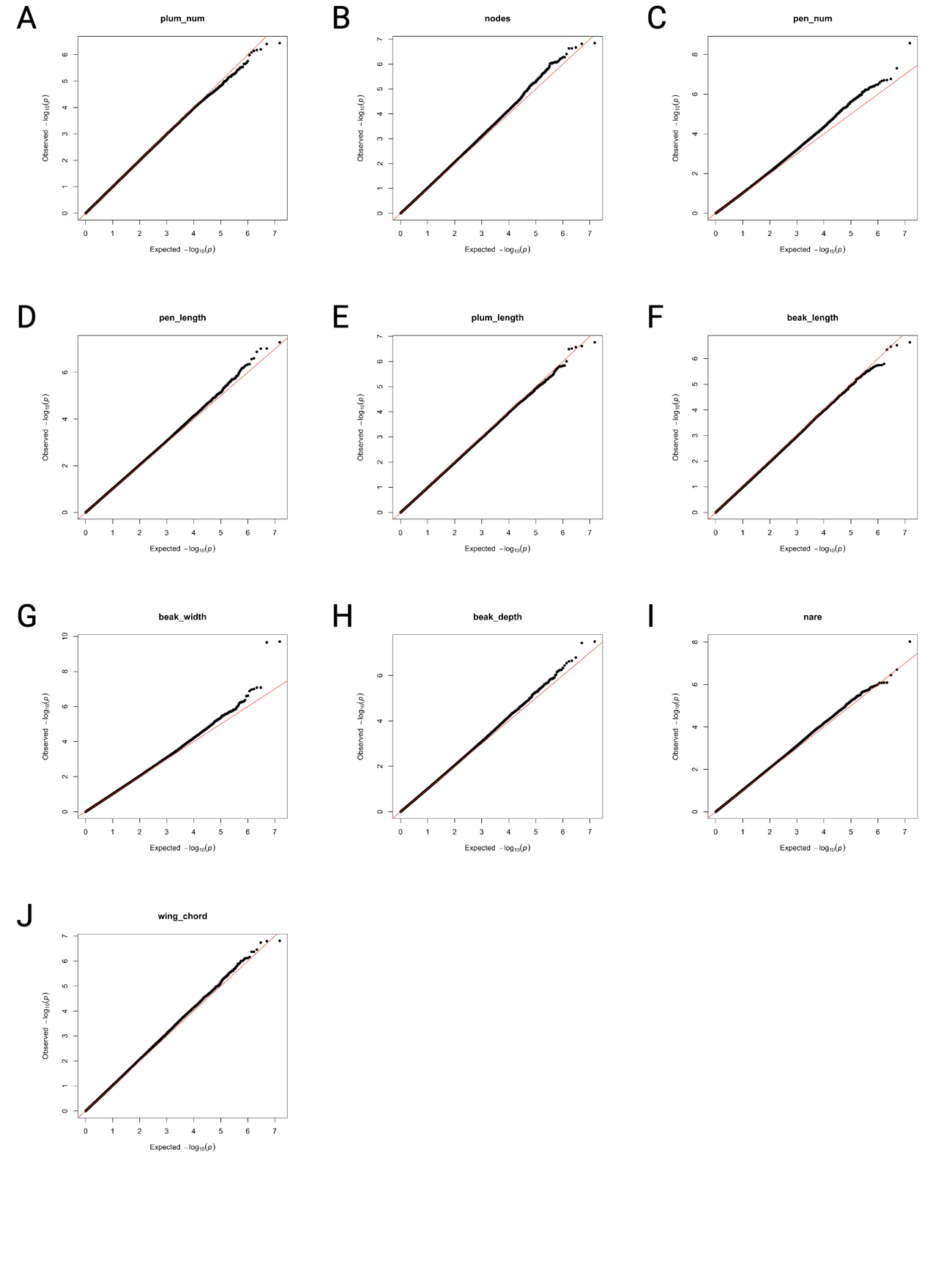

Supplement: Supplementary file 1 — Data S1: ece372962‐sup‐0001‐Supinfo.zip. [file ECE3-16-e72962-s001.zip › ece372962-sup-0001-Supinfo.docx]
